# Liquid–liquid phase separation facilitates the biogenesis of secretory storage granules

**DOI:** 10.1083/jcb.202206132

**Published:** 2022-09-29

**Authors:** Anup Parchure, Meng Tian, Danièle Stalder, Cierra K. Boyer, Shelby C. Bearrows, Kristen E. Rohli, Jianchao Zhang, Felix Rivera-Molina, Bulat R. Ramazanov, Sushil K. Mahata, Yanzhuang Wang, Samuel B. Stephens, David C. Gershlick, Julia von Blume

**Affiliations:** 1 Department of Cell Biology, Yale University School of Medicine, New Haven, CT; 2 Cambridge Institute for Medical Research, University of Cambridge, Cambridge, UK; 3 Departments of Pharmacology and Neuroscience, Fraternal Order of Eagles Diabetes Research Center, University of Iowa, Iowa City, IA; 4 Internal Medicine, Division of Endocrinology and Metabolism, University of Iowa, Iowa City, IA; 5 Department of Molecular, Cellular and Developmental Biology, University of Michigan, Ann Arbor, MI; 6 Department of Neurology, University of Michigan School of Medicine, Ann Arbor, MI; 7 Department of Medicine, University of California San Diego, La Jolla, CA; 8 VA San Diego Healthcare System, San Diego, CA

## Abstract

Insulin is synthesized by pancreatic β-cells and stored into secretory granules (SGs). SGs fuse with the plasma membrane in response to a stimulus and deliver insulin to the bloodstream. The mechanism of how proinsulin and its processing enzymes are sorted and targeted from the trans-Golgi network (TGN) to SGs remains mysterious. No cargo receptor for proinsulin has been identified. Here, we show that chromogranin (CG) proteins undergo liquid–liquid phase separation (LLPS) at a mildly acidic pH in the lumen of the TGN, and recruit clients like proinsulin to the condensates. Client selectivity is sequence-independent but based on the concentration of the client molecules in the TGN. We propose that the TGN provides the milieu for converting CGs into a “cargo sponge” leading to partitioning of client molecules, thus facilitating receptor-independent client sorting. These findings provide a new receptor-independent sorting model in β-cells and many other cell types and therefore represent an innovation in the field of membrane trafficking.

## Teaser

The biogenesis of secretory granules (SGs) is poorly understood. Here, we show that chromogranin proteins form a liquid scaffold upon entry into the milieu of the trans-Golgi network (TGN). These scaffolds recruit clients such as proinsulin and facilitate their transport to SGs.

## Introduction

Secretory proteins control human metabolism and physiology, and their mistargeting causes several diseases, including type 2 diabetes (T2D), neurodegenerative disorders, and cancer (Uhlén et al., 2019; Frantz et al., 2010). Regulated secretion is a process in which cells store signaling molecules in secretory storage granules which are large vesicular structures found in endocrine, exocrine, and neuronal cells, and then release their content in response to a stimulus (Burgess and Kelly, 1987; Miller and Moore, 1990).

Early electron microscopy imaging showed that secretory granules (SGs) contain an electron-dense core that is modified/concentrated in sequential steps starting in the Golgi apparatus (Orci et al., 1987; Palade, 1975; Farquhar et al., 1978; Farquhar and Wellings, 1957), wherein granule-destined proteins are sorted and packaged into short-lived vesicular intermediates known as immature SGs (ISGs; Tooze et al., 1991). Understanding the sorting and targeting of proteins from the Golgi apparatus to secretory storage granules in professional secretory cells such as insulin-secreting pancreatic β islets remains a challenge in the field.

Secretory proteins are synthesized, glycosylated, folded, and quality checked in the endoplasmic reticulum (ER) lumen before being loaded onto carriers for transport to the Golgi apparatus (Brandizzi and Barlowe, 2013; Dancourt and Barlowe, 2009; Miller and Barlowe, 2010). These secretory proteins travel across the Golgi stacks and are sorted at the trans-Golgi network (TGN; Ford et al., 2021). The TGN sorts and targets proteins to their destination through vesicular transport carriers (Kienzle and von Blume, 2014; Ramazanov et al., 2021). Importantly, proinsulin sorting to ISGs must be tightly coordinated with its processing by Carboxypeptidase E (CPE), Proprotein convertase 1/3 (PC1/3), and PC2 (Hutton, 1989; Germanos et al., 2021; Arvan, 2004). Remarkably, proinsulin and its processing enzymes do not have a conserved structural or sequence-specific motif for sorting them into ISGs, and a cargo receptor has not yet been identified (Bauerfeind and Huttner, 1993; Thiele et al., 1997; Arvan and Halban, 2004; Chung et al., 1989).

Chromogranin (CG) proteins are highly expressed in pancreatic β-cells and other specialized secretory cells and play a significant role in the packaging of granule-destined proteins (Bearrows et al., 2019; Kim et al., 2001; Tooze and Huttner, 1990). Previous studies suggest that CG aggregation in the TGN may be critical for concentrating these molecules in the TGN lumen (Tooze and Huttner, 1990; Chanat and Huttner, 1991). Ectopic expression of either CGA or CGB induces SG-like structures in non-secretory cells (Huh et al., 2003; Kim et al., 2001; Rustom et al., 2002; Wacker et al., 1997). Experiments using isolated granule contents from adrenal or pituitary glands and purified CGA, CGB, or secretogranin II (SCG II) from vesicle lysates of bovine adrenal medullary chromaffin cells demonstrated aggregation of these proteins at an acidic pH (5.2–5.5; Colomer et al., 1996; Yoo, 1995; Gerdes et al., 1989) and high (millimolar) calcium concentrations (Gerdes et al., 1989; Colomer et al., 1996; Yoo, 1995; Chanat and Huttner, 1991). Importantly, these conditions did not match with the physiological milieu of the TGN lumen (Paroutis et al., 2004; Pizzo et al., 2011), and so, the physiological relevance of CG aggregation in sorting remained unclear. Aggregation of CGs was determined by analyzing the proteins in pellet fractions upon centrifugation or by turbidity measurements. Hence, a detailed biophysical characterization of higher ordered assemblies of CGB as well as its connection to cargo recruitment has remained elusive. Therefore, the identity and function of CG aggregates in cells remained unknown.

Liquid–liquid phase separation (LLPS) is increasingly recognized as a major principle of macromolecular organization that concentrates certain components and promotes specific biochemical reactions (Shin and Brangwynne, 2017; Alberti et al., 2019; Alberti, 2017). The formation of these condensates is usually driven by multivalent interactions among proteins. De-mixing of a homogenous solution gives rise to a dense and a dilute phase with exchange of molecules between the two phases (Alberti and Hyman, 2021). On the other hand, protein aggregation refers to a higher order irreversible protein assembly. These can arise due to interactions between unfolded proteins and are associated with pathological conditions (Alberti and Hyman, 2021). One commonly identified element associated with phase-separating proteins is the intrinsically disordered region (IDR), an amino acid sequence characterized by low sequence complexity (Forman-Kay and Mittag, 2013; Martin and Holehouse, 2020). However, in some instances, proteins with well-structured domains can also undergo LLPS (Schwarz-Romond et al., 2005; Bienz, 2020). Simple charge patterns and the overall sequence composition of the IDRs are critical for the phase separation (Schuster et al., 2021). Other crucial factors controlling LLPS are the concentration and identity of macromolecules as well as the conditions of the microenvironment surrounding the condensates including pH and ionic concentration.

Here, we show CGB undergoes LLPS in the TGN lumen in INS1 832/13 cells and in vitro and the CGB condensates recruit clients like proinsulin. While the mildly acidic pH is a trigger for CGB LLPS, divalent cations like calcium are dispensable. Importantly, the material properties of CGB are critical for client recruitment as CGB condensates engage clients but aggregates fail to do so. Unlike previously thought, client recruitment to the CGB condensates is largely nonspecific and dependent upon the abundance of cargo clients at the TGN. CGB condensation is driven by the intrinsically disordered N-terminal domain of CGB. Finally, we observed that CGB condensation and proinsulin recruitment facilitate granule biogenesis and secretion of mature insulin. With these results, we have established the molecular basis of proinsulin sorting at the TGN in pancreatic β-cells, a so far unresolved question in cell biology.

## Results

### CGB and CGA undergo LLPS in vitro at an acidic pH resembling the TGN milieu

**Figure 1.**
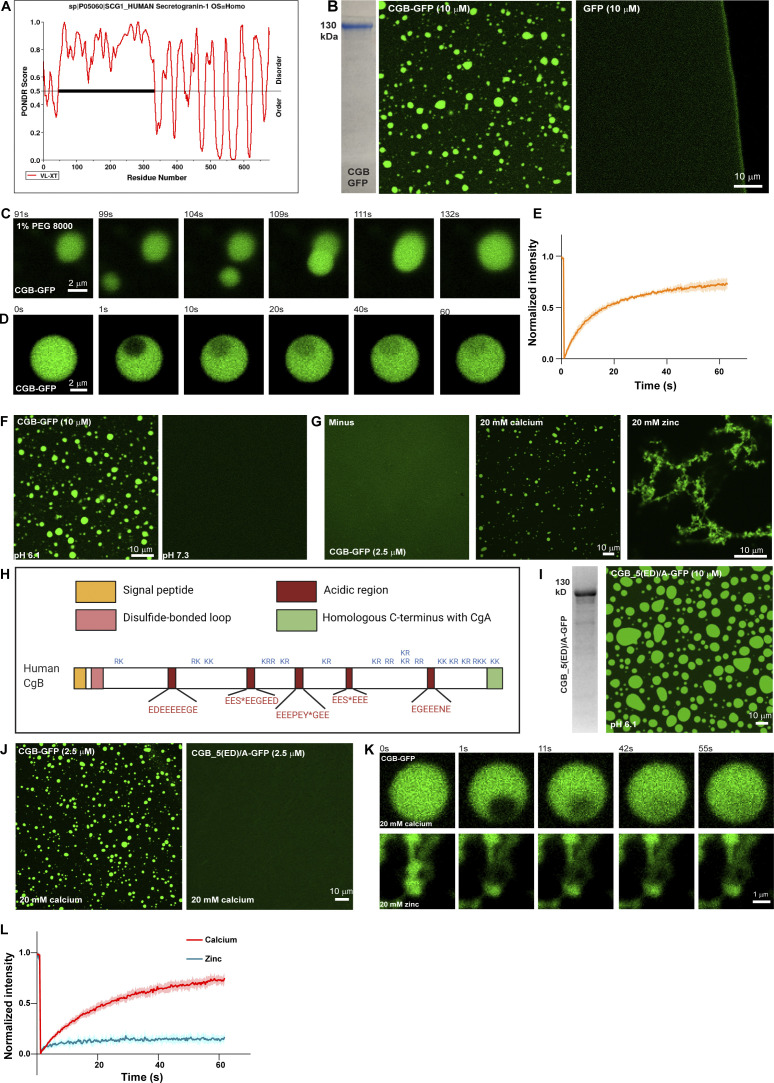
**CGs undergo LLPS in vitro at a mildly acidic pH, while calcium is dispensable. (A)** Plots of CGB generated using PONDR depicting disordered regions in the proteins. N-terminal half of CGB is completely disordered, when analyzed using the VL-XT algorithm. **(B)** Coomassie -stained gel depicting purified CGB-GFP. The images show droplets of CGB-GFP (left) at 10 µM protein concentration at pH 6.1 without a crowding agent. The same concentrations of super folder-GFP do not form droplets at pH 6.1 (right). **(C)** A panel of images extracted from a movie showing the biophysical behavior of CGB-GFP (2.5 µM) droplets induced by 1% PEG 8000. Two droplets, which come in proximity undergo fusion and the larger droplet subsequently relaxes into a spherical shape. **(D)** A panel of images monitoring recovery of fluorescence of CGB-GFP droplets after bleaching a small region within the droplets. Note the rapid recovery of fluorescence (more than 60%) in the bleached region within a minute. **(E)** Graph quantifying the fluorescence recovery in time. Data represented as mean ± SD (error blanket) from seven droplets. **(F)** Representative images of solutions containing CGB-GFP (10 µM) buffered at either pH 6.1 (left) or pH 7.3 (right). Droplet formation occurs at pH 6.1 and not at pH 7.3. **(G)** Representative images of CGB-GFP (2.5 µM) protein without any divalent cation (minus) or in presence of 20 mM calcium, and zinc, respectively. CGB-GFP solution (∼10 µM concentration) was centrifuged to preclear of existing droplets and diluted to a final concentration of 2.5 µM. While calcium induces CGB-GFP droplets, zinc induces formation of insoluble aggregates. **(H)** Schematic representation of CGB, depicting five exons. Patches in red are stretches enriched in acidic amino acids. S* and Y* denote phosphorylation and sulfation respectively on these residues. KR or RK are the dibasic sites in the protein which are predominantly concentrated in the C-terminal half of the protein. **(I)** Representative Coomassie-stained image of mutant form of CGB, CGB_5(ED)/A-GFP to depict its purity. In this mutant the 5 (ED) stretches have been replaced with alanine. The fluorescence image shows condensates of CGB_5(ED)/A when equilibrated at pH 6.1 at 10 µM protein concentration. **(J)** Comparison of CGB-GFP and CGB_5(ED)/A-GFP in presence of 20 mM calcium concentrations. Note that at 2.5 µM protein concentration, calcium can induce droplet formation with only CGB-GFP (left), but now with CGB_5(ED)/A-GFP (right). **(K and L)** Snapshots captured at different time points after photobleaching a region within a calcium-induced CGB-GFP droplet (top) or a zinc induced CGB-GFP aggregate (bottom) are shown in (K). While there is more than 60% fluorescence recovery within the bleached region in the calcium induced droplet, recovery within the bleached region in the aggregate is only 10% as seen in the graph in (L). Red curve denotes recovery of calcium-induced droplets and blue curve denotes zinc-induced aggregates. Data is represented as mean ± SD (error blanket) from seven calcium-induced droplets and six zinc-induced aggregates. Source data are available for this figure: SourceData F1.

**Figure S1.**
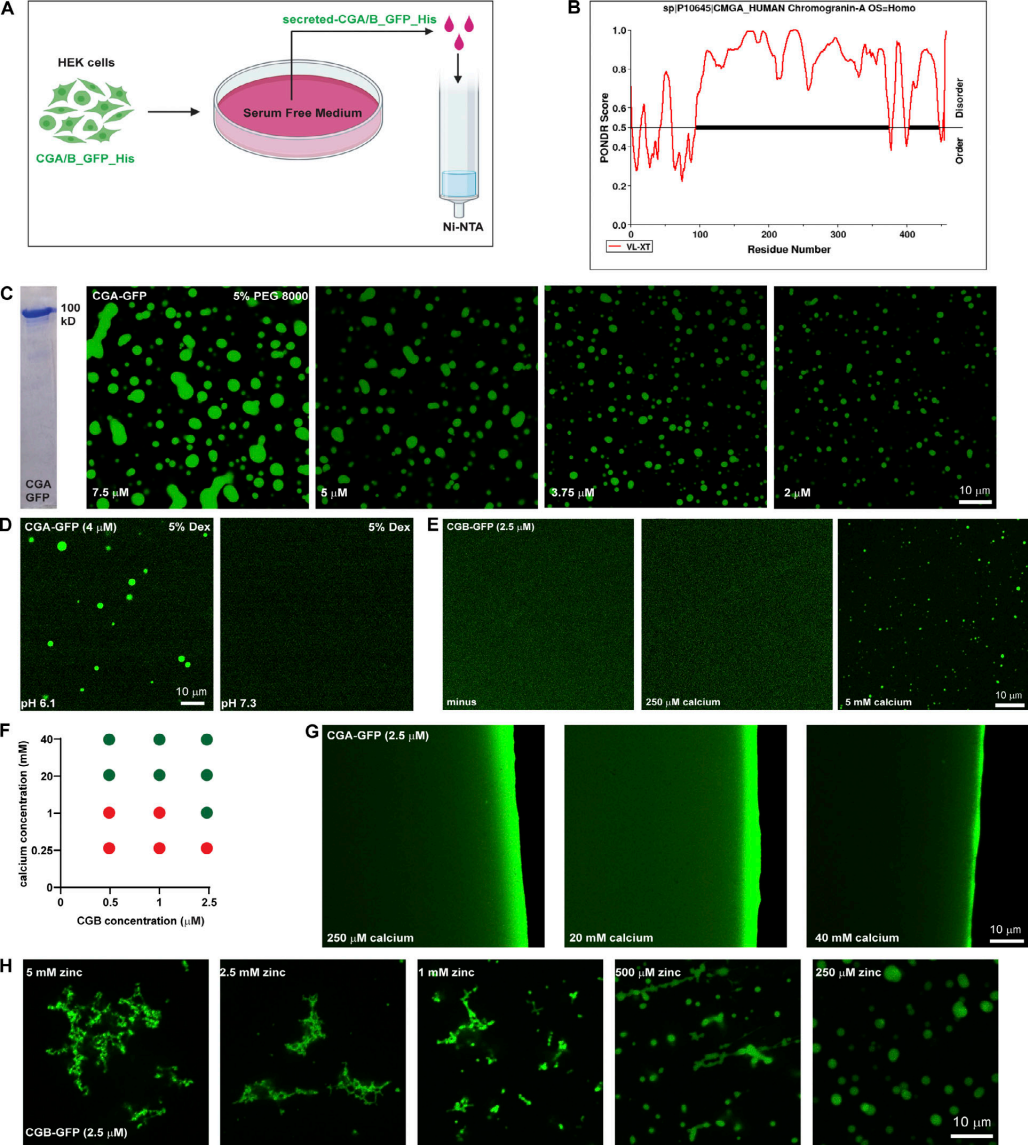
**In vitro characterization of CG LLPS. (A)** Scheme used for purifying GFP and 6x-His-tagged CGA or CGB, respectively. Stable lines expressing CGB-GFP and CGA-GFP under a doxycycline-inducible promoter are treated with doxycycline and the calcium ionophore A23187 to induce secretion of the respective proteins in serum-free medium, which is then used for purification using Ni-NTA affinity columns. **(B)** Plot of CGA generated using PONDR depicting disordered regions in the proteins. Almost 90% of CGA is disordered when analyzed using the VL-XT algorithm. **(C)** Coomassie-stained gel depicting purified CGA-GFP. Images showing different droplets of CGA-GFP at different protein concentrations at pH 6.1 in presence of 5% PEG 8000. Note that the size of the condensates decreases with decreasing protein concentration. **(D)** Representative images of solutions containing CGA-GFP buffered at either pH 6.1(left) or pH 7.3 (right). Droplet formation occurs at pH 6.1 and not at pH 7.3. Droplets of CGA-GFP were induced at 4 µM protein concentration in presence of 5% dextran. **(E)** Images obtained from plating a solution of CGB-GFP (2.5 µM final concentration) to monitor the presence or absence on liquid-like condensates either without or with 250 µM or 5 mM calcium. Note that droplet formation is induced only in the presence of 5 mM calcium. **(F)** Phase diagram obtained by varying calcium and CGB concentrations after plating solutions on imaging dishes and observing after 15–20 min. Red circles indicate conditions where no droplets were seen, and green circles indicate conditions which favored presence of CGB droplets. **(G)** Images obtained from plating a solution of CGA-GFP (2.5 µM final concentration) in presence of either 250 µM, 20 or 40 mM calcium to test for the presence or absence of droplet formation. No droplets are seen even at 40 mM which is the highest calcium concentration. **(H)** Representative images of CGB-GFP (2.5 µM) in presence of different concentrations of zinc. At high concentrations zinc induces formation of insoluble aggregates but at low concentration, it induces CGB-GFP droplets. Source data are available for this figure: SourceData FS1.

Members of the Granin family of proteins have been shown to aggregate at an acidic pH and in the presence of millimolar concentrations of calcium (Yoo, 1995; Chanat and Huttner, 1991; Colomer et al., 1996). However, the biochemical conditions used in the assays do not match the TGN, which has a mildly acidic pH around 6.1 and 130 µM calcium concentration at steady state (Pizzo et al., 2011; Paroutis et al., 2004). Therefore, the biophysical nature and the mechanism of aggregation remained unclear. To gain an insight into the molecular basis of CG aggregation, we first analyzed CGA or CGB sequences using the database PONDR (http://www.pondr.com). PONDR predicted that 50% of CGB and 90% of CGA are intrinsically disordered (Fig. 1 A and Fig. S1 B), one of the features associated with proteins shown to undergo LLPS. We thus tested whether purified CGs form liquid-like condensates. To this end, we collected the cell culture supernatants of HEK293 cells stably expressing His-tagged CGA or CGB, respectively, C-terminally fused to monomeric super folder GFP (sfGFP). His-tagged proteins were purified using Ni-NTA columns (Fig. S1 A). Purified proteins were obtained with high purity (Fig. 1 B and Fig. S1 C). To determine whether CGs form condensates, we incubated CGs in a buffer with physiological salt concentration and pH 6.1 resembling the TGN milieu (Paroutis et al., 2004). For visualization, we plated either CGA-GFP or CGB-GFP protein solutions onto an imaging dish and analyzed it by fluorescence microscopy. We observed CGB condensates/droplets, whereas sfGFP remained soluble under the same conditions (Fig. 1 B). In contrast to CGB, purified CGA-GFP in solution did not form condensates when equilibrated at pH 6.1. Nevertheless, we could induce condensate formation of CGA-GFP by adding PEG 8000, a routinely used crowding agent to the protein solution (Fig. S1 C). The size of the condensates was dependent on protein concentration. Upon decreasing the protein concentration, the condensate sizes decreased (Fig. S1 C).

Next, we used time-lapse imaging of CGB-GFP droplets to determine whether they exhibit liquid-like properties. Two or more condensates in proximity display fusion and relaxation to a spherical shape (Fig. 1 C). Moreover, when we bleached a small region within the CGB condensate, more than 50% of the fluorescence recovered within 1 min, indicating high mobility of proteins within the condensates (Fig. 1, D and E). These data demonstrated that purified CGs undergo LLPS in vitro and that CGB has a higher potential for phase separation than CGA.

Secretory proteins are exposed to changes in the biochemical environment (milieu) as they travel through the sub-compartments of the biosynthetic pathway. Therefore, we next investigated the impact of pH on CG-LLPS by testing the role of the intrinsic pH difference between the ER (approximate pH 7.3) and TGN (approximate pH 6.1) compartments (Paroutis et al., 2004). Notably, we observed that the pH of the solution was critical for the reaction because condensation only occurred at pH 6.1, not at pH 7.3, consistent with the TGN pH (Fig. 1 F and Fig. S1 D). These results demonstrated that CG condensates are formed in a pH resembling the TGN milieu suggesting a compartment-specific triggering mechanism for CG condensation.

### Calcium is dispensable for LLPS of CGs

CGs have been shown to aggregate in the presence of high calcium concentrations (Chanat and Huttner, 1991; Colomer et al., 1996; Yoo, 1995). Therefore, we hypothesized that calcium would promote CG-LLPS. To test this, purified CGA or CGB at pH 6.1 was centrifuged and then diluted to 2.5 µM concentration at which we did not observe any droplets (Fig. S1 E). We then tested for droplet formation by varying calcium concentration in the buffer. The steady state of free calcium in the TGN is estimated to be around 130 µM (Pizzo et al., 2011). Surprisingly, however, CGA or CGB condensates did not form at 250 µM of calcium which is closer to the estimated physiological concentration (Fig. S1, E and F). CGB LLPS could be induced at 5 mM calcium and increased at 20 mM calcium (Fig. 1 G and Fig. S1 E). We further tested droplet formation of CGB as a function of varying concentrations of calcium and protein and found that calcium-induced LLPS of CGB was favored at high CGB protein and calcium concentrations (Fig. S1 F). At 2.5, 1, and 0.5 µM CGB concentrations (where protein does not undergo LLPS on its own), droplet formation was seen at millimolar calcium concentrations, but not close to physiological calcium levels (250 µM; Fig. S1 F). As previously shown in Fig. 1 B, at higher protein concentrations, CGB condensation happens even in the absence of calcium. CGA failed to form droplets even with calcium concentrations as high as 40 mM (Fig. S1 G). Although CGB binds to calcium (Schmidt et al., 2011), no structural binding motif, such as an EF-hand domain, has been identified. CGB is rich in acidic amino acid residues and a striking feature of the CGB protein is five clusters of contiguous negatively charged amino acids. Therefore, we hypothesized that these charge patches could facilitate calcium binding to CGB at high calcium concentrations. To test this hypothesis, we mutated all acidic residues in the five negatively charged patches to alanine (CGB_5(ED)/A) and expressed and purified the protein as described previously. Next, we analyzed condensation with and without calcium by fluorescence microscopy. While CGB_5(ED)/A formed droplets at 10 µM protein concentration, without calcium (Fig. 1 I), comparable to the wild-type protein, in contrast, CGB_5(ED)/A did not condense in the presence of 20 mM calcium (Fig. 1 J). These findings establish that the five patches enriched in negatively charged residues are calcium-binding sites in CGB while also further ruling out the necessity for calcium in driving CGB LLPS. In summary, we showed that calcium promotes CGB condensation at high concentrations in millimolar range, which are not seen in the TGN lumen at steady state. Contrary to the current paradigm, CGB condensates form in the absence of calcium and thus calcium is dispensable for the reaction.

As we never observed CGB aggregates in the presence of calcium, we tested the impact of other divalent cations playing a role in the secretory pathway of insulin-secreting cells. Zinc is essential for hexamerization of mature insulin (Dodson and Steiner, 1998) and is present at high concentrations (mM levels) in SGs. To test if zinc has an impact on CGB-LLPS, we incubated CGB-GFP in the presence of 20 mM zinc. Interestingly, zinc induced the formation of filamentous aggregates (Fig. 1 G). To test the dynamicity of these aggregates, we bleached a small region of interest and monitored the recovery rate (Fig. 1, K and L). Importantly, the CG aggregates did not recover after photobleaching, demonstrating the loss of its dynamicity and LLPS behavior. Interestingly, upon titrating the zinc concentrations, we observed a mixture of droplets and aggregates at 1 mM zinc and droplets at a lower concentration (Fig. S1 H). Thus, high concentrations of zinc (aggregates) or calcium (liquid-like condensates) generate CGB assemblies with different material properties and provide a powerful tool to investigate the significance of the biophysical state of CGB for sorting and packaging granule-destined proteins.

### SG-targeted cargo is organized in dynamic punctate structures at the Golgi apparatus

**Figure 2.**
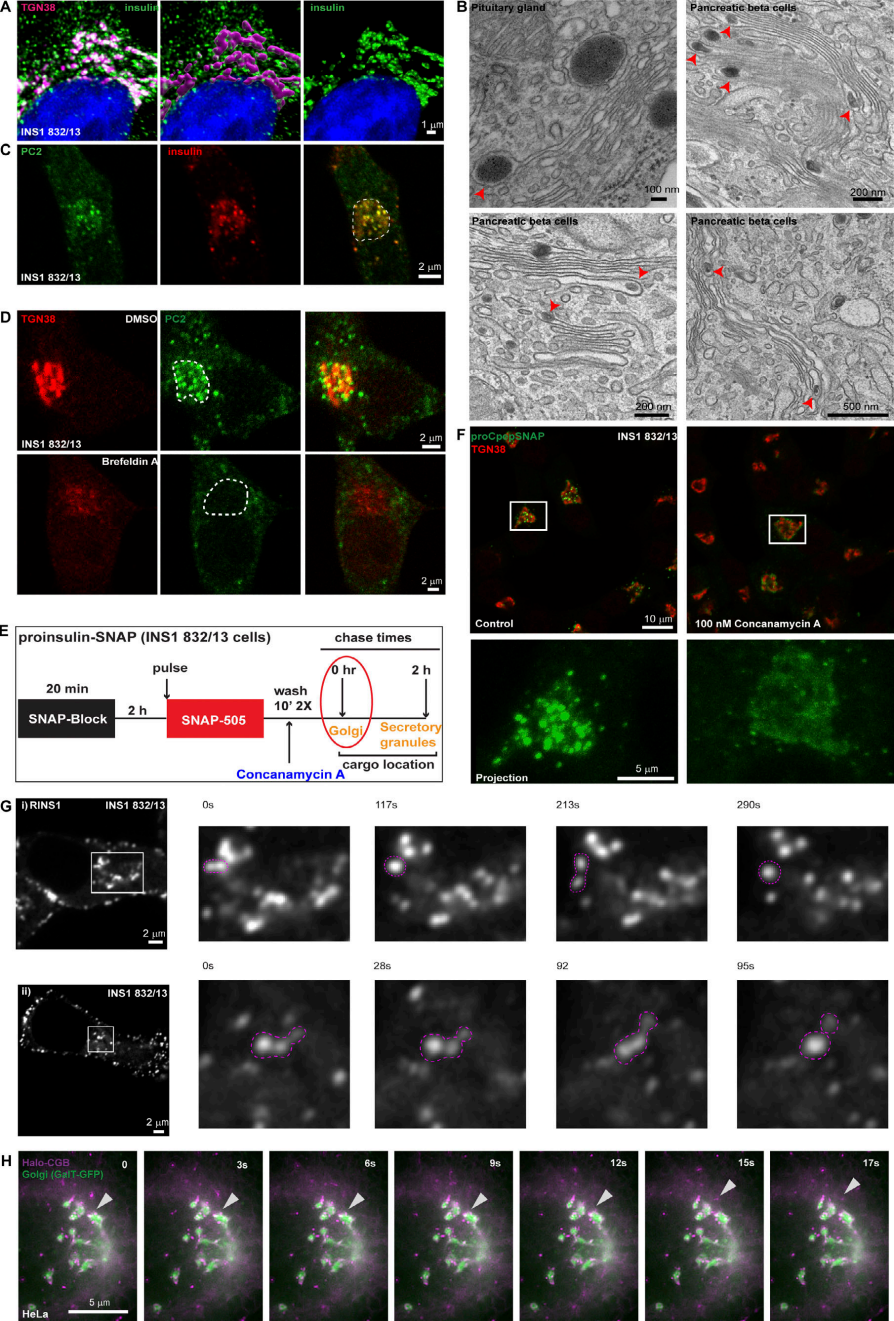
**Insulin is distributed in dynamic punctate structures at the Golgi apparatus. (A)** INS1 832/13 cells were immunostained for insulin (pan-insulin antibody; green), TGN38 (magenta) and counterstained with DAPI (blue). The middle image demonstrates 3D-rendering of the TGN38 volume mask which was used for image segmentation to specifically examine insulin staining within the TGN (right image). **(B)** A panel of electron micrographs from cells of pituitary gland and mouse islets demonstrating condensed structures within the Golgi lumen. **(C)** Representative images from a single slice from a confocal image of INS1 832/13 cells stained with the insulin processing enzyme protein convertase 2 (PC2; green) and insulin (pan-insulin antibody; red). Note the colocalization of proinsulin puncta with PC2 at the Golgi apparatus which is outlined in the merge image using dashed lines. **(D)** Images obtained from INS1 832/13 cells which were stained with TGN38 (red) and PC (green), treated with either 0.05% DMSO (control; top) or 5 µg/ml Brefeldin A (bottom). Shown here is a single slice from a confocal stack. Note the disassembly of TGN38 based on staining upon Brefeldin A treatment which is accompanied by a loss of PC2 puncta in the perinuclear region, marked using dashed line. **(E)** A schematic description of a pulse-chase assay in INS1 832/13 cells stably expressing SNAP-tagged proinsulin. Cells are initially incubated with a non-fluorescent blocking probe to mask the existing proteins in the cells. After 2 h, cells are labeled with SNAP-505 to mark the newly synthesized proteins (20 min). After two washes in growth medium either in presence or absence of concanamycin A (control), cells are fixed immediately to monitor the pulse of new synthesized proinsulin arriving at the Golgi apparatus. **(F)** Representative images at the top show single slice from a confocal image of INS1 832/13 cells labeled with SNAP-505 (green) to monitor newly arrived proinsulin at the TGN (red) in control (left) and concanamycin A (right) treatment. The insets below are zoomed in images from a single cell to highlight the punctate vs diffuse distribution of proinsulin at the TGN in control (left) and concanamycin A (right) treatment. **(G)** A panel of images extracted from a movie from live imaging of INS1 832/13 cells transiently transfected with RINS1, a fluorescent insulin reporter construct. Dynamics of the punctate structures (pink dashed line) are captured in the image sequence where structures undergo fission or fusion events. Images shown here are single confocal slices upon imaging in the conventional confocal mode (i) or in the airy scan confocal mode (ii). Images have been smoothened using the function in ImageJ for visual representation purposes. **(H)** A panel of images extracted from a movie from live imaging of HeLa cells expressing Halo-RUSH-CGB (magenta) and GalT-GFP (green) to monitor the budding of CGB granules from the Golgi. The arrowhead denotes a budding event from the Golgi.

The results obtained with our in vitro assays showed that CGs phase separate depending on the pH of the solution. Next, we wanted to assess if these condensates are present in cells and if their biophysical properties match with the in vitro observations. To this end, we analyzed insulin localization in a cell-culture model of pancreatic β-cells (INS1 832/13) by immunofluorescence microscopy and pancreatic islets using transmission electron microscopy (t-EM). We observed that proinsulin occurs in punctate structures at the Golgi apparatus (Fig. 2 A). We reconstructed the volume of the TGN using 3D-rendering of confocal stacks from cells labeled using TGN38 and pan-insulin antibodies. Within the volume mask representing the TGN lumen, insulin staining is distinctly punctate (Fig. 2 A). We further confirmed the presence of electron-dense structures budding from within the Golgi stack through t-EM of mouse islets as well as in cells of the pituitary gland (Fig. 2 B). Proinsulin processing occurs during granule maturation (Fu et al., 2013), and processing enzymes must be co-sorted into ISGs to facilitate proper insulin maturation. To test whether proteolytic enzymes colocalize with proinsulin puncta, we labeled the cells with pan-insulin and protein convertase 2 (PC2) antibodies. We observed that PC2 colocalized with the proinsulin puncta in the Golgi apparatus (Fig. 2 C).

Incubation of cells with Brefeldin A (BFA) has been shown to result in disassembly of the Golgi apparatus and translocation of the associated proteins to the ER (Alcalde et al., 1992). Treatment of INS1 832/13 cells with BFA resulted in a significant change in the staining pattern of TGN38 (Fig. 2 D), indicating disassembly of the TGN. Importantly, this was accompanied by the disappearance of the perinuclear PC2 puncta implying these structures are associated with the Golgi apparatus and are not post-Golgi vesicles.

**Figure S2.**
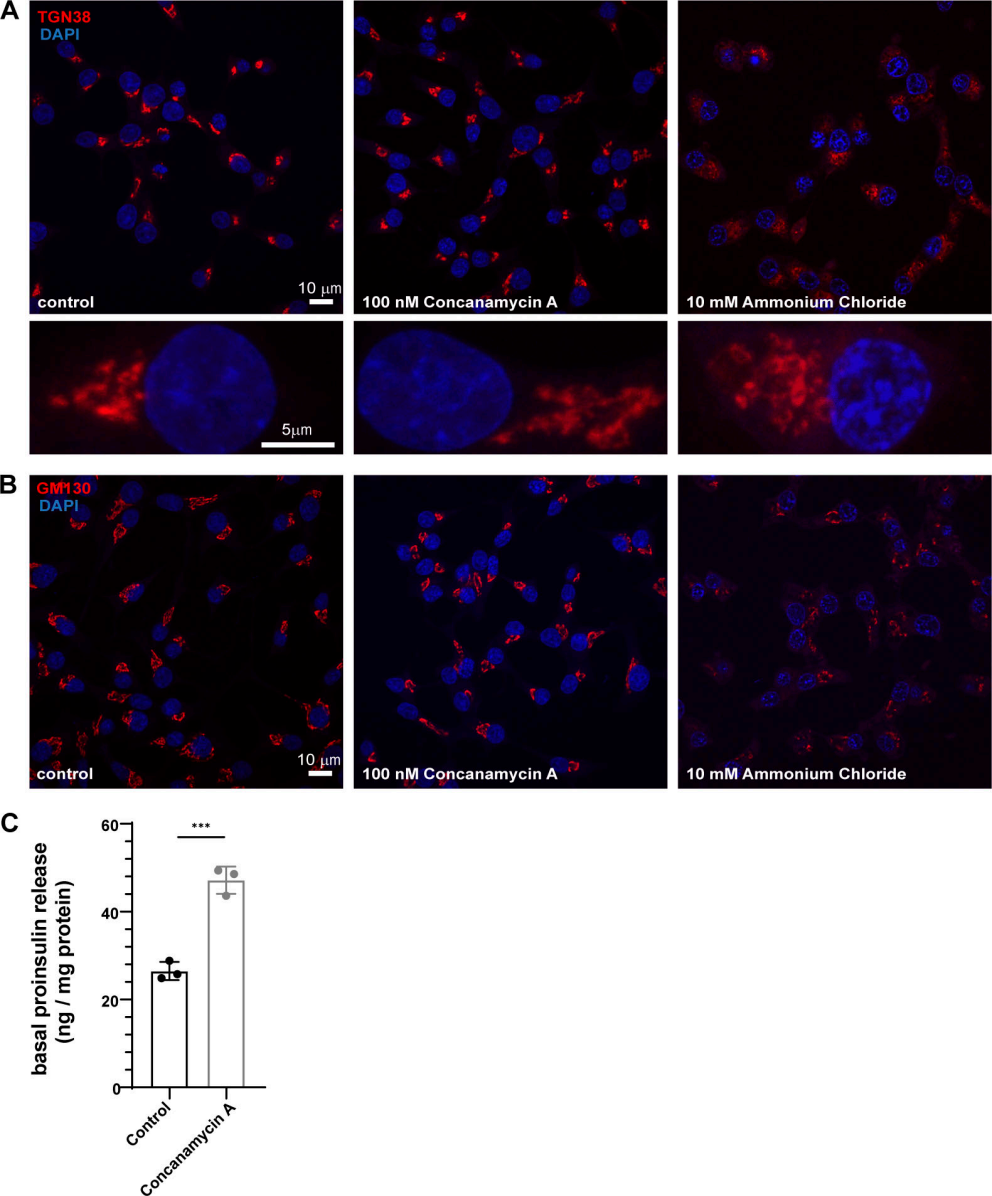
**Effects of concanamycin A on organization of Golgi apparatus and proinsulin secretion from INS1 832/13 cells. (A and B)** INS1 832/13 cells fixed and labeled with antibodies to TGN38 in A; red, and GM130 in (B); red in control (left), 100 nM concanamycin A treatment for 20 min (middle) or 10 mM ammonium chloride treatment for 20 min (right). The rectangular boxes in A represent projection of zoomed images from a single cell. Not that while TGN38 staining looks similar to control cells upon concanamycin A treatment, it appears expanded and fragmented upon ammonium chloride treatment. The staining intensity is reduced upon ammonium chloride treatment and hence the scaling is not same for representation purposes. **(C)** Graph showing normalized proinsulin secretion (proinsulin secreted/total protein in cells) at steady state in presence of 3 mM glucose (basal) to monitor the secretion of proinsulin via the constitutive pathway in INS1 832/13 cells with and without concanamycin A treatment. Data is represented as mean ± SD from three independent experiments. Statistical analysis was performed using unpaired two-tailed *t* test. ***P < 0.001.

Based on the role of mildly acidic pH governing the in vitro LLPS of granins, we investigated the effects of alkalinization of the TGN pH using concanamycin A, a known inhibitor of the V0-V1 ATPase. Concanamycin A treatment acutely elevates the pH of the Golgi apparatus (Schapiro et al., 2000). We utilized the previously established SNAP-tagged proinsulin (Bearrows et al., 2019) to probe its distribution in the presence of concanamycin A. The SNAP-tagged proinsulin has been shown to be secreted in response to high glucose and potassium chloride (KCl), like the wild-type protein (Bearrows et al., 2019) and confirmed in this study (Fig. 4 H). To test the effects of pH, we introduced concanamycin A during washes after incubation of SNAP-505 probe (Fig. 2 E). While we observed proinsulin in punctate structures in the TGN in control cells, proinsulin showed a diffuse pattern in the presence of concanamycin A (Fig. 2 F; and Videos 1 and 2). We also monitored the morphology of the Golgi apparatus by staining for TGN38 and GM130, which appeared normal in concanamycin A treatment, but it was perturbed upon incubation of cells with 10 mM ammonium chloride for 20 min (Fig. S2, A and B). We further tested if the punctate distribution of proinsulin at the Golgi apparatus impacts secretion of proinsulin by monitoring its basal secretion (3 mM glucose), which is anticipated to occur through the constitutive pathway. Interestingly, we observed increased secretion of proinsulin upon concanamycin A treatment (Fig. S2 C) implying leaky secretion through the constitutive pathway due to a failure to be routed to SGs.

**Video 1.** **3D projections of INS1 832/13 cells expressing SNAP tagged insulin and labeled using SNAP505 to monitor the newly arrived pool at the Golgi apparatus in control cells.** Scale bar is 5 µm.

**Video 2.** **3D projections of INS1 832/13 cells expressing SNAP tagged insulin and labeled using SNAP505 to monitor the newly arrived pool at the Golgi apparatus in cells treated with 100 nM concanamycin A.** Scale bar is 5 µm.

To visualize the dynamic nature of proinsulin puncta in the TGN, in situ INS1 832/13 cells were transfected with RINS1, a dual fluorescently tagged form of proinsulin where mCherry is inserted within the C-peptide and sfGFP is present at the C-terminus (Schifferer et al., 2017). Cells were then monitored using time-lapse live-cell imaging. The proinsulin puncta in the Golgi apparatus displayed occasional fusion when in proximity, indicating liquid-like behavior in vivo (Fig. 2 G). Overall, the observations revealed that proinsulin is organized in dynamic punctate structures at the Golgi apparatus.

Next, to visualize the budding of CGB from the TGN, we applied the Retention Using Selective Hooks (RUSH) system in which cargo release from the ER can be controlled by addition of biotin to the cell-culture supernatant (Boncompain et al., 2012). Ectopic expression of granins in non-secretory cells induces the formation of ectopic SGs (Huh et al., 2003; Kim et al., 2001; Rustom et al., 2002; Wacker et al., 1997). As RUSH trafficking assays in HeLa cells are very well established, we used this cell model for the next approach. HeLa cells expressing Halo-RUSH-CGB and the TGN marker GalT-GFP were incubated with biotin and the release of CGB from the ER toward the cell surface was imaged in live cells using lattice-structured illumination imaging (lattice-SIM). We observed motile puncta of CGB at the Golgi apparatus which bud off into the cytoplasm (Fig. 2 H and Video 3). Collectively using these approaches, we demonstrated that CGB and proinsulin are organized in punctate, condensate-like structures in living cells and display dynamic mobility and behavior akin to LLPS at the TGN.

**Video 3.** **Live imaging of HeLa cells expressing Halo-RUSH-CGB (magenta) and GalT-GFP (green) to monitor the dynamics and budding of CGB structures from the Golgi apparatus.** Imaging was carried out after biotin addition when the CGB had reached the Golgi apparatus. Scale bar is 5 µm.

### Proinsulin and CGB are co-sorted into TGN-derived transport carriers

**Figure 3.**
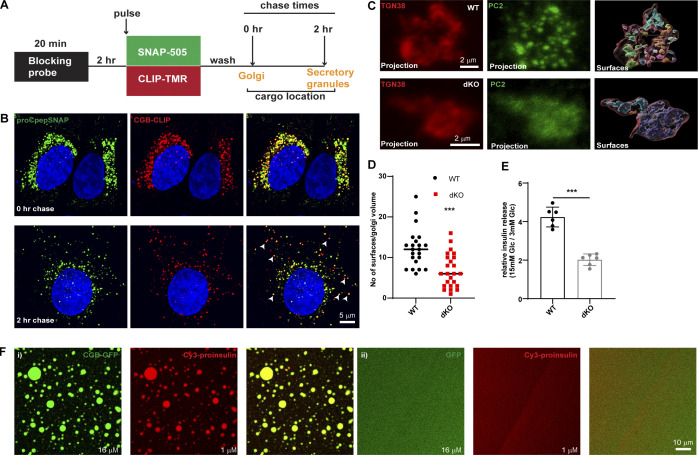
**Proinsulin co-traffics with CGB in vivo and is recruited to droplets in vitro. (A)** Schematic depiction of dual pulse chase experiment in INS1 832/13 cells expressing SNAP tagged insulin (proCpepSNAP) and CLIP tagged CGB (CGB-CLIP). Cells are initially incubated with a non-fluorescent blocking probe to mask the existing proteins in the cells. After 2 h, cells are incubated with medium containing SNAP 505 and CLIP-TMR to label the newly synthesized proteins (20 min). After three washes in growth medium, cells are fixed immediately (0 h chase) when majority of the cargo is at the Golgi apparatus or after a chase of 2 h where most of the cargo has moved to the SG in the cytoplasm. **(B)** Top panel shows confocal images form INS1 832/13 cells expressing SNAP tagged insulin (proCpepSNAP; green) and CLIP tagged CGB in red and fixed immediately after labeling with fluorescent probes, SNAP-505 and CLIP-TMR to monitor the Golgi resident (peri-nuclear) pool of the proteins. Bottom panels show images after a 2 h chase and the arrows point to some of the colocalizing structures which are cytoplasmic SGs. **(C)** INS1 832/13 wild-type (top) and CGA/CGB dKO (bottom) cells fixed and labeled with antibodies to TGN38 (red) and PC2 (green). Left and the middle images are extracted from a 3D projection. The image on the right represents surfaces which were created using the TGN38 staining (red outline) on deconvolved images in Imaris. The TGN38 volume mask was then used to generate distinct surfaces in the PC2 channel. **(D)** A scatter plot (median) depicting differences in the numbers of PC2 surfaces between wild-type and CGA/CGB dKO cells from 22 wild-type and 24 dKO cells. Statistical analysis was performed using Mann–Whitney test. ***P < 0.001. **(E)** Graph showing normalized glucose stimulated insulin secretion (GSIS; stimulated/basal) in wild-type, CGA/CGB dKO cells. Data is represented as mean ± SD from six independent experiments. Statistical analysis was performed using unpaired two-tailed *t* test. ***P < 0.001. **(F)** CGB-GFP (16 µM; green) was mixed with Cy3 tagged proinsulin (1 µM; red) in (i). Tagged proinsulin gets recruited to the CGB-GFP droplets as evident from the colocalization image. When GFP (16 µM; green) is mixed with Cy3 tagged proinsulin (1 µM; red) in (ii), no droplets are seen either with GFP or proinsulin indicating that GFP or Cy3-proinsulin are incapable of forming droplets on their own at these concentrations.

**Figure S3.**
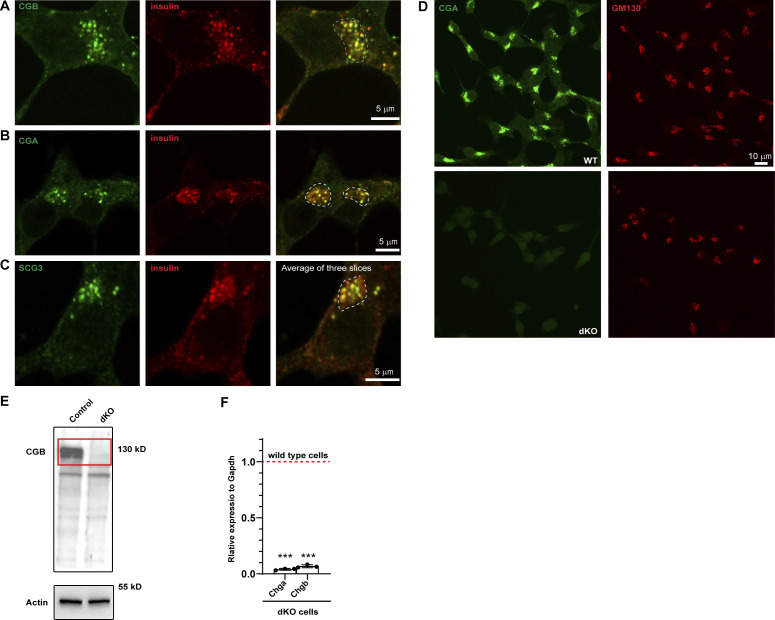
**Localization of endogenous granin proteins with insulin in INS1 832/13 cells and validation of CGA/CGB double KO cells. (A–C)** INS1 832/13 cells fixed and labeled with antibodies to insulin in or CGB (A), CGB (B) and SCG III (C) in green. Insulin puncta at the Golgi apparatus in the perinuclear region, outlined using the dashed lines in the merge colocalize with each of the granin proteins. For CGB and CGA, images are a single slice from a confocal stack and in case of SCG III, an average projection from three consecutive slices from a confocal image. **(D)** Representative images of INS1 832/13 cells (wild type; top) and CGA/CGB double knockout (dKO; bottom) stained for CGA and GM130 antibodies to validate absence of CGA staining from the dKO cells. **(E)** Western blot (top) shows cell lysates from INS1 832/13 wild-type and dKO cells probed using CGB antibody. Note the absence of band in dKO cells which have been highlighted using the red rectangle. The bottom blot is probing of the same membrane for actin, which is used as a loading control. **(F)** qPCR to monitor the reduction in transcripts for Chga and Chgb in wild-type and dKO cells. Values are represented as mean ± SD from three independent experiments and expressed relative to Gapdh. Relative values for wild-type cells are normalized to 1. Statistical analysis was performed using unpaired two-tailed *t* test ***P < 0.001. Source data are available for this figure: SourceData FS3.

Based on the previously described evidence for CGB condensation, we hypothesized that CG-LLPS is a necessary step for the sorting of proinsulin at the TGN. Depletion of CGB in INS1 832/13 cells results in impaired proinsulin export from the TGN (Bearrows et al., 2019). If CGB condensation sorts proinsulin in the TGN, both proteins should colocalize and bud into the same transport carrier exiting the TGN. To address this, we established a pulse-chase approach to monitor the simultaneous export of proinsulin and CGB from the TGN in living cells. We expressed CLIP-tagged CGB in INS1 832/13 cells stably expressing SNAP-tagged proinsulin (Fig. 3 A). Newly synthesized SNAP-proinsulin and CLIP-CGB entering the TGN were labeled with their respective fluorescent analogs (*t* = 0) and their colocalization was analyzed (Fig. 3 B). Following a 2-h chase of the respective fluorescent SNAP or CLIP probes, both proteins colocalized in cytoplasmic granules, indicating that they are sorted into the same transport carriers exiting the TGN (Fig. 3 B). Moreover, at steady state insulin showed colocalization with CGB, CGA, and secretogranin III (SCG III) in the TGN, TGN-derived vesicles, and cytoplasmic granules (Fig. S3, A–C).

To gain further insights into the relationship between granin proteins, proinsulin, and other SG-directed cargo, we generated a CGA/CGB double knockout (dKO) cell line using CRISPR/Cas9 (Fig. S3, D–F). To analyze the distribution of the SG-directed cargo at the TGN, we dually stained the control and the dKO cells using antibodies to TGN38 and PC2. Like proinsulin, the distribution of PC2 occurs in a punctate pattern at the TGN. However, in dKO cells, it occurs in a diffuse pattern further demonstrating that CGB condensation is required for PC2 droplet formation at the TGN in vivo (Fig. 3, C and D). More importantly, the dKO cell line exhibited a substantial reduction of glucose-stimulated insulin secretion (GSIS; Fig. 3 E). Thus, CG-driven LLPS at the TGN is functionally important in vivo for cargo sorting and secretion of mature insulin.

Can proinsulin be recruited to CGB condensates in vitro? To address this question, we applied our well-established in vitro system mimicking TGN conditions. CGB-GFP (16 µM final concentration) and Cy3-tagged proinsulin (1 µM final concentration) were combined at pH 6.1. Recruitment of proinsulin to the CGB condensates was readily observed (Fig. 3 F); no co-condensation of GFP and proinsulin occurred (Fig. 3 F). Based on these findings, we propose that CGs co-condense along with proinsulin and its processing enzymes in the luminal milieu of the TGN, resulting in its export from the TGN for its secretion in a regulated manner.

### Ectopic expression of constitutively secreted proteins results in their targeting to SGs and co-secretion with insulin

**Figure 4.**
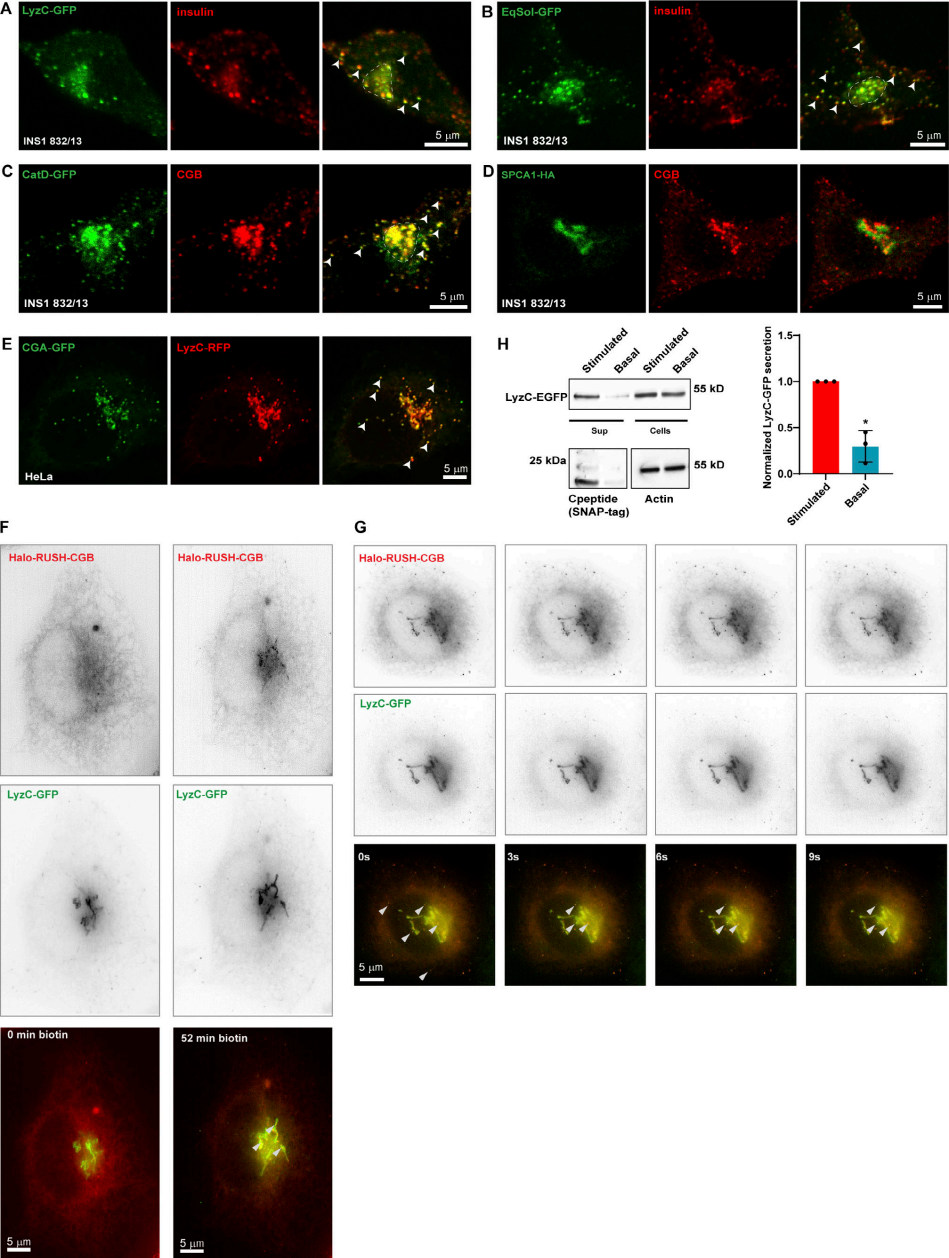
**Ectopic expression of soluble secreted proteins in INS1 832/13 cells results in their routing to insulin granules. (A and B)** Representative images from INS1 832/13 cells expressing LyzC-GFP (A; green) or EqSol-GFP (B; green) and stained with insulin antibody (red) to observe the localization of the ectopically expressed proteins with respect to insulin granules. Images are average projections from two slices from a confocal stack. Arrowheads point to cytoplasmic insulin granules which also shows the presence of LyzC-GFP and EqSOL-GFP respectively, in E and F. **(C)** Representative images from INS1 832/13 cells stably expressing CatD-GFP (green) and labeled with CGB antibody to observe the localization of ectopically expressed CatD-GFP with respect to SGs. Images are average projections from two slices from a confocal stack. Arrowheads point to some of the cytoplasmic SG, which shows colocalization of CGB and CatD-GFP. **(D)** Representative images from INS1 832/13 expressing HA-tagged version of the calcium ATPase, SPCA1 (green) and stained using CGB antibody (red). Images are a single slice from a confocal stack. Note that overexpressed SPCA1 remains localized at the Golgi apparatus with no signal seen from the CGB containing SGs. **(E)** Representative images from HeLa cells stably expressing CGA-GFP and transfected with LyzC-RFP. Images are a single slice from a confocal stack imaged in the airy-scan mode. The arrowheads point to some of the ectopic granule-like structures seen in HeLa cells upon expression of CGA-GFP. Note that LyzC-RFP gets routed to these ectopic granule-like structures. **(F)** Images extracted from live imaging of HeLa cells co-expressing Halo-RUSH-CGB (red) and LyzC-GFP (green) before and after addition of biotin for 52 min when CGB appears at the Golgi. **(G)** Images extracted from live imaging of HeLa cells co-expressing RUSH-CGB (red) and LyzC-GFP (green) after biotin addition and images after arrival of CGB at the Golgi. Arrow heads point to colocalizing structures at the Golgi and vesicles in the cytoplasm. **(H)** Western blot at the top shows bands for LyzC-GFP, probed using α-GFP antibody, in supernatant and lysates from INS1 832/13 cells stable expressing SNAP-tagged proinsulin. The basal condition represents cells grown in 3 mM glucose in serum-free medium and the stimulated condition represents cells grown in 15 mM glucose in serum-free medium, also containing 35 mM potassium chloride. Note the stronger band intensity in the supernatant in stimulated condition compared to the basal condition, although the levels in cell lysates are the same. The blot in the bottom left detects the presence of SNAP-tagged C-peptide, probed using α-SNAP-tag antibody, which is used as a proxy to measure insulin secretion. Again, the signal intensity of the band is stronger in stimulated condition as compared to the basal condition. The blot on the bottom right depicts actin bands in cell lysates obtained from basal and stimulated conditions. The graph quantifies secretion of LyzC-GFP normalized with levels in cell lysates in basal and stimulated conditions. Value of the band intensity in secreted compared to the band intensity in cell lysates was set to 1 for stimulated condition in each experiment. Data is represented as mean ± SD from three independent experiments. Statistical analysis was performed by two-tailed one-sample *t* test *P = 0.019. Source data are available for this figure: SourceData F4.

**Figure S4.**
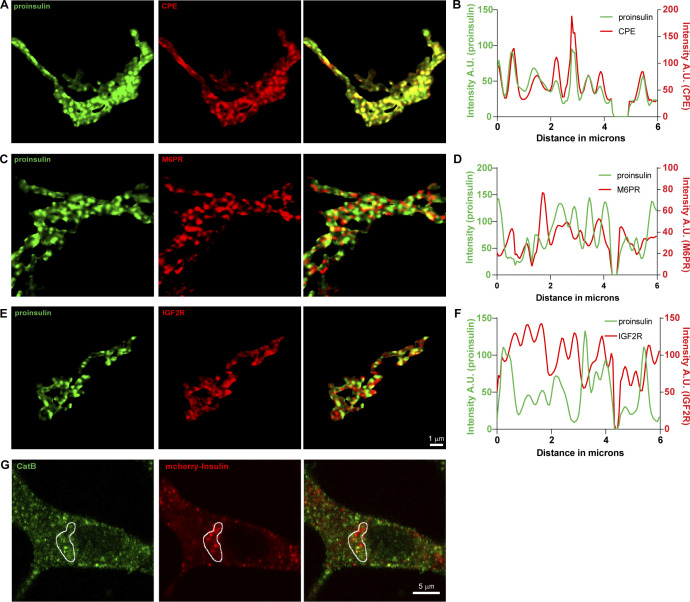
**Localization of receptors for lysosomal hyrolases, Cathepsin B and insulin at the Golgi and cytoplasm in INS1 832/13 cells. (A–F)** INS1 832/13 cells were immunostained for insulin (pan-insulin antibody; green), TGN38 (not shown) and CPE (A), M6PR (C), or IGF2R (E). **(A, C, and F)** Image segmentation of the TGN38 volume was used to identify Golgi-staining regions. **(B, D, and F)** Line intensity profiles of the Golgi staining regions for each protein are shown. **(G)** Single slice from a confocal image obtained from INS1 832/13 cells expressing mCherry and Apex tagged proinsulin (red) and stained with an antibody against Cathepsin B (green). Area outlined in white denotes the Golgi region which was marked using GM130 staining which is not shown here.

Next, we asked whether constitutively secreted proteins segregate from proinsulin-CG droplets, which would align with the “sorting for entry” model (Tooze, 1998). In non-professional secretory cells, Cab45 sorts a subset of proteins, including lysoyzyme C (LyzC), to the cell surface (von Blume et al., 2012). Therefore, proinsulin localization was compared with that of ectopically expressed constitutively secreted LyzC-GFP or EqSol-GFP (point mutation in Equinatoxin which abolishes binding to sphingomyelin)—a non-secretory protein that has been used as a soluble bulk flow marker when designed to enter the secretory pathway (Deng et al., 2016). Unexpectedly, we observed colocalization of these proteins with proinsulin, and both LyzC-GFP and EqSol-GFP were transported to SGs in insulin-secreting cells (Fig. 4, A and B). We then analyzed localization of the ectopically expressed lysosomal hydrolase cathepsin D (CatD) in INS1 832/13 cells. In contrast to an endogenously expressed hydrolase (Fig. S4 G), ectopically expressed CatD-sfGFP was not targeted to the lysosome but instead routed to insulin granules (Fig. 4 C). Importantly, at the TGN, proinsulin is segregated from receptors for lysosomal hydrolases, Mannose 6-phosphate receptor and IGF2R (Fig. S4, C–F). Consistent with previous research (Hummer et al., 2020), routing of cargoes to SGs appears to be limited to soluble proteins, as ectopic expression of HA-tagged SPCA1, a transmembrane protein localized to the TGN, did not result in its routing to SGs but instead SPCA1 remained Golgi-localized (Fig. 4 D). To further investigate whether routing to SGs depends on CGs, we expressed RFP-tagged LyzC and CGA-sfGFP in HeLa cells, which are naturally deficient in CGs and SGs. We detected ectopic SGs in HeLa cells expressing CGA-sfGFP and observed the routing of LyzC-RFP to these granules (Fig. 4 E).

To further investigate recruitment of LyzC to CGB condensates in living cells, we co-expressed LyzC-GFP and Halo-RUSH-CGB in HeLa cells. For time-lapse imaging, we used lattice-SIM after addition of biotin, mediating synchronous release of CGB from the ER whereas LyzC-GFP is distributed throughout ER and Golgi consistent with the distribution of a constitutively secreted protein in HeLa cells (Deng et al., 2018). Consistent with the data shown in Fig. 2 H, Halo-RUSH-CGB traffics from the ER to the Golgi apparatus, where it buds into motile post-Golgi carriers (Fig. 4 F). Importantly, upon TGN entry of CGB, we observed colocalization and active budding of LyzC-containing CGB carriers from the TGN (Fig. 4 G and Video 4). These results demonstrate direct client recruitment to CGB condensates in vivo and indicate that CG condensates can recruit client proteins independent of a conserved sequence for their identity or a sequence-encoded recognition signal. Importantly, CG expression overrides existing pathways for vesicular targeting needed for constitutive secretion.

**Video 4.** **Live imaging of HeLa cells expressing Halo-RUSH-CGB (red) and LyzC-GFP (green) demonstrates colocalization of both the proteins in dynamic punctate structures at the Golgi apparatus as well as in cytoplasmic granules.** Scale bar is 5 µm.

Since overexpression of secreted soluble proteins leads to their routing to the SGs in INS1 832/13 cells, we assessed whether they are also secreted in response to a glucose stimulus (Hohmeier et al., 2000). Indeed, at basal levels (3 mM glucose), we observed a minimal amount of basal secretion in cells expressing the constitutively secreted protein LyzC-GFP and SNAP-tagged C-peptide (proxy for insulin secretion). However, upon glucose (15 mM) and KCl (35 mM) stimulation of INS1 832/13 cells, both LyzC-GFP and insulin were co-secreted (Fig. 4 H). Thus, targeting of LyzC to CGB condensates at the TGN not only leads to its delivery to the SGs but also to its enhanced secretion along with insulin. Our data suggest that there does not appear to be a retrieval mechanism for mistargeted exogenous proteins.

### Constitutively secreted proteins co-segregate with CGB condensates in vitro

**Figure 5.**
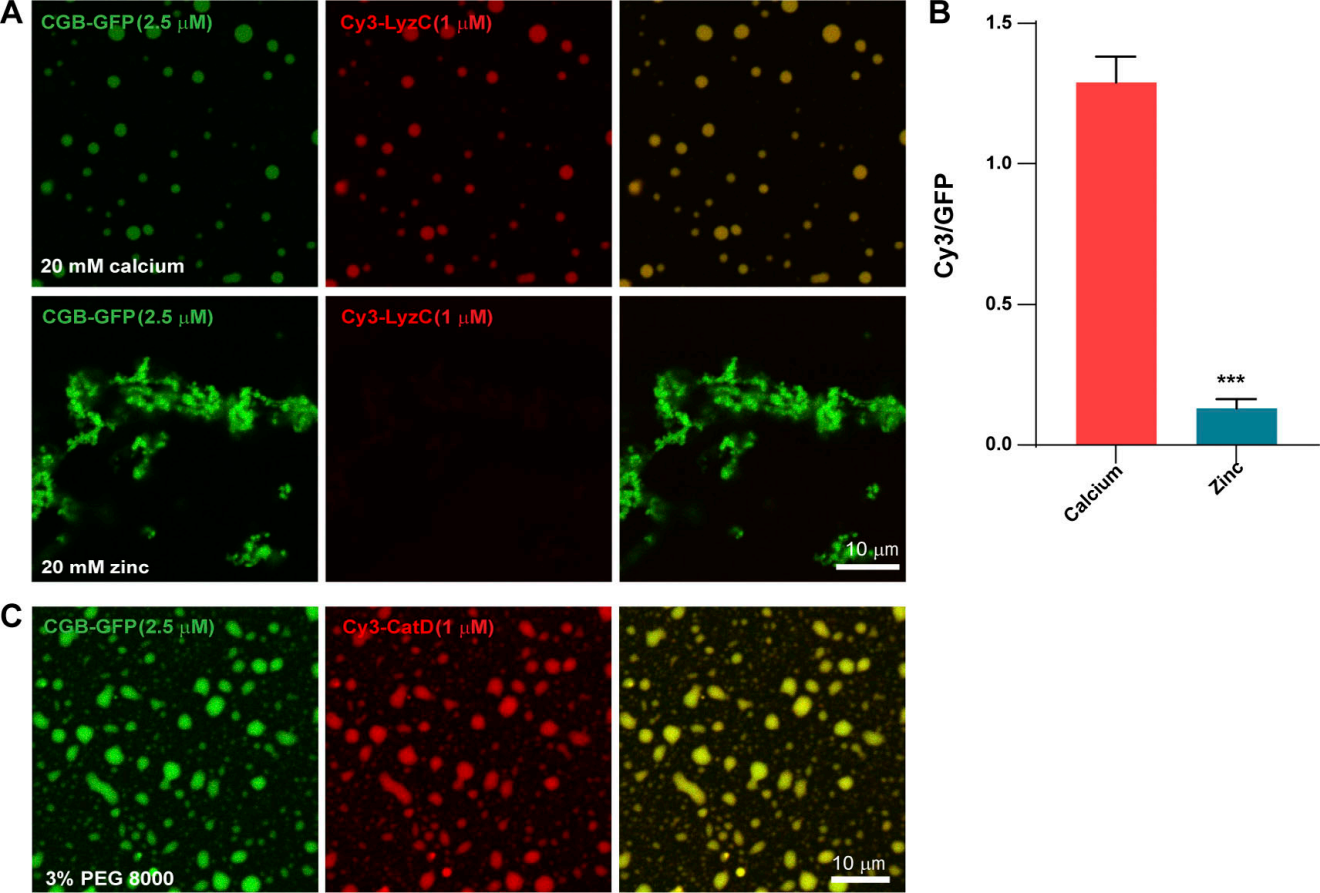
**Client recruitment in vitro. (A and B)** CGB-GFP (2.5 µM; green) was mixed with Cy3-tagged LyzC (1 µM; red) and was subsequently followed by addition of 20 mM calcium to induce liquid-like droplets or with 20 mM zinc to induce aggregates. While calcium-induced CGB-GFP droplets (top) recruit Cy3-LyzC, the zinc-induced aggregates (bottom) fail to recruit it. Bar graph is B quantifies the ratio of intensities in Cy3 v/s GFP channel, imaged at the same acquisition settings, to monitor recruitment of LyzC in relation to amount of CGB in the droplet (red) v/s aggregate (blue). Data is represented as mean ± SD from 28 droplets and 27 regions within aggregates. Statistical analysis was performed by unpaired two-tailed *t* test. ***P < 0.001. **(C)** CGB-GFP (2.5 µM; green) was mixed with Cy3-tagged CatD (1 µM; red) and droplet formation was induced using 3% PEG 8000 to monitor the recruitment of clients into the condensates. Note that Cy3-CatD shows strong recruitment to the droplets.

Based on routing of ectopically expressed LyzC-GFP, CatD-GFP, and EqSol-GFP to SGs, we wanted to determine whether these proteins get recruited to CGB condensates in vitro and whether liquid-like or solid aggregates of CGB have any differences in their ability to recruit clients. To test this at comparable concentrations of CGB and clients, CGB and Cy3-tagged LyzC were combined at 2.5:1 molar ratio and calcium and zinc were used to induce droplets and aggregates, respectively. As demonstrated earlier (Fig. 1, K and L), calcium induces condensation of CGB with properties similar to those displayed by CGB condensates formed without calcium. Cy3-LyzC was recruited to calcium-induced CGB condensates (Fig. 5, A and B). However, in contrast to the current view that aggregated CGs recruit clients, we did not detect Cy3-LyzC recruitment into the zinc-containing CGB aggregates (Fig. 5, A and B). In a similar experiment, we confirmed that Cy3-tagged CatD (routed to SGs upon overexpression in INS1 832/13 cells) was also recruited to the CGB condensates (Fig. 5 C). Together, these results highlight the importance of material properties of CGB assemblies for client recruitment and lack of a common sorting sequence in CGB clients.

### Truncation of CGB impacts phase separation potential and the ability to generate ectopic granules

**Figure 6.**
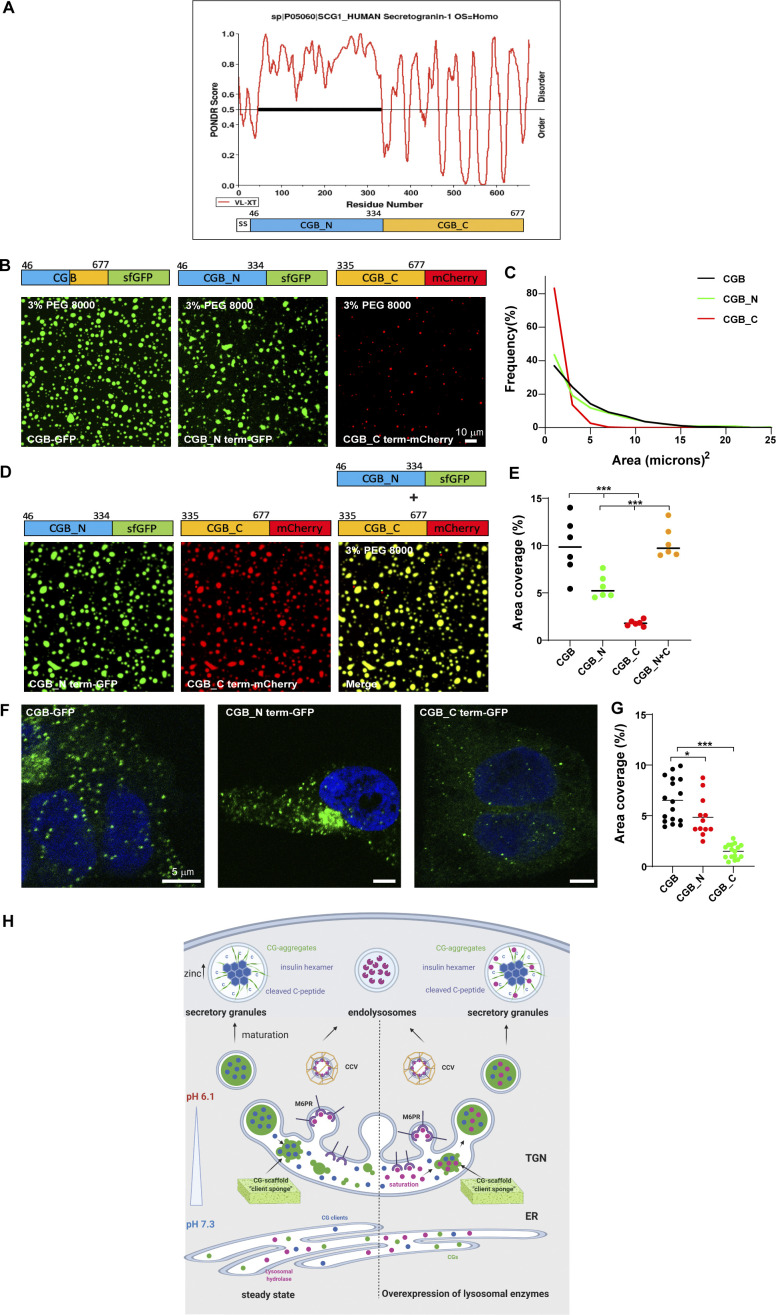
**In vitro and in vivo phenotypes associated with truncation mutants of CGB. (A)** Plots of CGB generated using PONDR (VL-XT algorithm) depicting disordered regions in the protein. Based on the PONDR scores, amino acids 46–334 are highly unstructured, thus showing a high degree of disorder compared to residues 335–677. **(B)** Representative images showing a comparison of condensates of full-length CGB-GFP, CGB_N term-GFP, and CGB_C term-mCherry, respectively. Droplet formation was initiated at pH 6.1 with 2 µM protein in the presence of 3% PEG 8000. **(C)** Histogram represents the frequency distribution of size of condensates for each of the three proteins. Area was quantified from 2610 droplets for CGB-GFP, 1450 droplets for CGB_N term-GFP, and 1716 droplets for CGB_C term-mCherry. Note that the size of the droplets is larger for CGB-GFP, CGB_N term-GFP compared to CGB_C term-mCherry. **(D)** Representative images of droplet formation obtained upon mixing CGB_N term-GFP (green) and CGB_C term-mCherry (red). Droplet formation was initiated at pH 6.1 upon mixing 2 µM protein of each protein in the presence of 3% PEG 8000. CGB_N term-GFP and CGB_C term-mCherry cooperatively form larger droplets together. **(E)** Scatter plot (median) quantifies area occupied by all the droplets in a field of view from a microscopy image. Data was pooled from 6 uniform fields of views. CGB-GFP > CGB_N term-GFP > CGB_C term-mCherry. Upon mixing CGB_N term-GFP with CGB_C term-mCherry, there is a restoration in the area covered by the condensates to the levels of the full-length protein. Statistical analysis was performed by unpaired two-tailed *t* test. ***P < 0.001. **(F)** Representative images of HEK293 cells expressing CGB-GFP (left), CGB_N term-GFP (middle) and CGB_C term-GFP (right) and induced with doxycycline for 10 h. Images were taken on a confocal microscope to observe ectopic granule-like structures in HEK293 cells, and the bottom-most plane of the cells plated on the coverslips was imaged. While CGB-GFP and CGB_N term-GFP expression induce formation of ectopic granules in HEK293 cells, much fewer granules are seen upon expression of CGB_C term-GFP. **(G)** Scatter plot (mean) quantifies the percentage area occupied by the ectopic granules as a fraction of total cell area. Data was obtained from 17 CGB-expressing cells, 12 CGB_N term-expressing cells and 16 CGB_C term-expressing cells pooled from two independent experiments. Statistical analysis was performed by unpaired two-tailed *t* test. *P = 0.043, ***P < 0.001. **(H)** Model presented summarizes our findings on the role of LLPS in receptor-independent cargo delivery from the TGN to the SGs. CGs undergo LLPS in the milieu of the TGN, behaving like a “cargo sponge” and recruit clients like proinsulin by the virtue of their relative abundance to the condensates. Lysosomal hydrolases would be sorted to the endolysosomes via the mannose-6-phosphate receptor pathway. However, upon overexpression, the receptors are saturated and hence owing to their smaller size and high abundance at the TGN, they get sucked into the CG condensates and hence delivered to the SGs. As the SGs mature, the presence of high zinc concentrations leads to hexamerization of insulin after its proteolysis. CGs can undergo aggregation and could represent one of the possible mechanisms for cargo segregation within the SGs.

**Figure S5.**
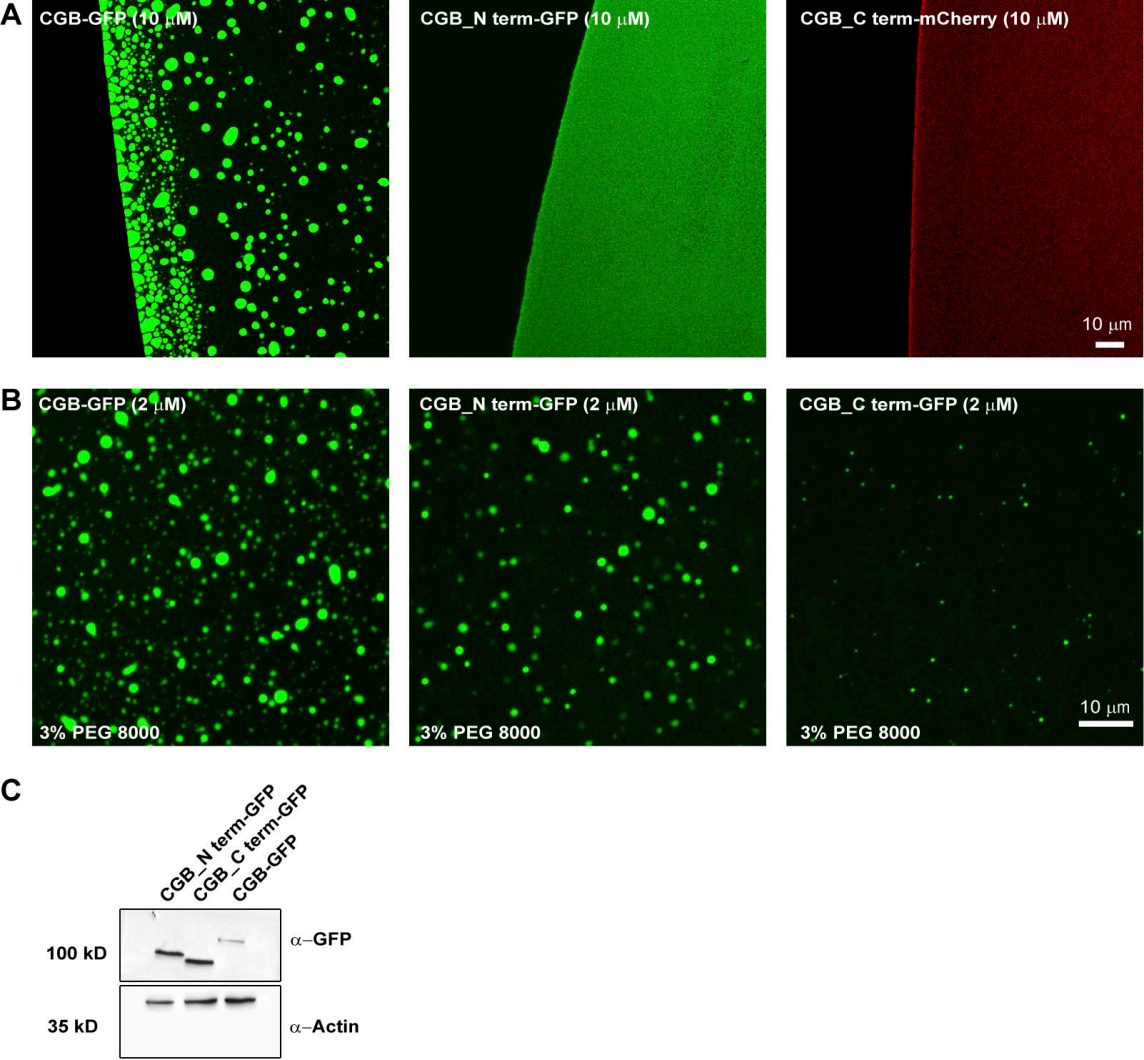
**LLPS capacity of truncation mutants of CGB. (A)** Images obtained by plating 10 µM of either CGB-GFP or CGB_Nterm-GFP or CGB_Cterm-mCherry without any crowding agent. Upon equilibration at pH 6.1 Note that droplet formation is seen only in CGB-GFP solution under these conditions. **(B)** Images obtained by plating of 2 µM of CGB-GFP or CGB_Nterm-GFP or CGB_Cterm-GFP in presence of 3% PEG 8000 to monitor droplet formation in these conditions. Only a few small droplets are observed in CGB_Cterm-GFP as compared to CGB-GFP or CGB_Nterm-GFP. **(C)** Western blots to compare the expression levels of CGB_Nterm-GFP and CGB_Cterm-GFP from HEK293 cells stably expressing these constructs upon induction using doxycycline and probed using GFP antibody (top). β-actin (bottom) was used as a loading control. Source data are available for this figure: SourceData FS5.

Decoding the information based on amino acid sequence in CGB which can drive condensate formation remains a challenge as many different factors contribute to LLPS (Schuster et al., 2021). IDR-containing proteins have been shown to undergo LLPS. The amino acid composition of IDRs combined with environmental factors seems to be major drivers of LLPS (Schuster et al., 2021). To determine if the disordered region of CGB impacts its phase separation, we truncated the protein into distinct parts based on its predicted structure (PONDR, http://www.pondr.com; Xue et al., 2010). The N-terminal domain of CGB (amino acids: 46–334 aa) is highly unstructured (high degree of disorder) compared to the C-terminal (amino acids: 335–667; Fig. 6 A). Hence, sfGFP- or mCherry-tagged truncation mutants of CGB, CGB_N term-GFP, and CGB_C term-mCherry were generated. For each mutant, the signal sequence of CGB was incorporated at the N-terminus to allow transit through the secretory pathway. Neither of the truncation mutants displayed condensate formation on its own at pH 6.1 (Fig. S5 A); however, upon addition of 3% PEG 8000, the N-terminal portion of CGB was observed to undergo a significantly more potent LLPS than that of the C-terminal (Fig. 6 B). There was a difference in area covered by the droplets observed for each mutant, with the droplets of CGB C-terminal fusion protein occupying the least area (Fig. 6, C and E). The inability of the CGB C-terminal to undergo LLPS was not due to the mCherry tag because the sfGFP-tagged version displayed the same phenotype (Fig. S5 B). Interestingly, when the N- and C-terminal truncation mutants were combined and phase separation was induced in the presence of 3% PEG 8000, the mixture displayed phase separation comparable to that of full-length CGB-GFP (Fig. 6, D and E). These results suggest that while most of the information responsible for mediating LLPS of CGB are encoded by the N-terminal domain, the C-terminal is required for optimal LLPS.

To test the effects of CG-LLPS on SG biogenesis, we took advantage of the CG protein characteristic of inducing ectopic granule-like structures when expressed in non-secretory cells (Beuret et al., 2004; Huh et al., 2003). For this purpose, we used stable HEK293 cell lines expressing CGB-GFP, CGB_N term-GFP, and CGB_C term-GFP, and induced expression using doxycycline. We observed that ectopic expression of both full-length and N-terminal CGB-induced biogenesis of ectopic granules, which were much lower when the C-terminal CGB was expressed (Fig. 6, F and G). Altogether these results reveal that the potential of CGB to undergo LLPS is directly linked to its ability to induce SG formation. Thus, CG LLPS is not only critical for sorting soluble proteins within the TGN lumen but also for driving SG biogenesis.

## Discussion

To investigate the molecular role of CGs in proinsulin sorting, we purified fully functional recombinant CGA and CGB, which are significant drivers of granule biosynthesis, in mammalian cells. We set up a microscopy-based in vitro assay to mimic the conditions in the TGN. We demonstrated that CGs undergo LLPS in conditions resembling the TGN milieu (pH 6.1) to form scaffolds for client recruitment. The pH in the TGN lumen is the primary driver of CG condensation and client (proinsulin) recruitment, whereas calcium is dispensable for the reaction. We also show that the material state of the condensates is crucial for client recruitment. Solid aggregates of CGB fail to recruit soluble clients, and truncation of CGB significantly affects its capacity to undergo LLPS. Furthermore, we show the abundance and dynamics of these condensates in living cells and confirm the significance of their formation for granule biogenesis and insulin secretion. Based on these findings, we propose that phase separating CGs act as a “client sponge,” incorporating clients based on their relative abundance within the TGN independent of their discrete sequence features.

### LLPS drives cargo condensation in the TGN

The predominant model that explains cargo sorting at the TGN in specialized secretory cells is termed “sorting for entry” or “sorting by aggregation” (Tooze, 1998), and has two main tenets: (i) granin family proteins undergo aggregation at an acidic pH and in the presence of calcium, after which the resultant aggregate, surrounded by a membrane, buds off from the TGN to form the ISG and (ii) granins aggregate and exclude proteins destined for lysosomes or constitutively secreted proteins, mediating cargo sorting toward the SG at the TGN. The molecular mechanism underlying generation for higher order assemblies of CGs under physiological conditions of the TGN lumen as well as specific cargo sorting by the aggregate remained unresolved. Furthermore, their identity in live cells remained unexplored.

Here by the virtue of being able to purify fluorescent-tagged versions of CGB and CGA and using a combination microscopy and biochemical methods, both in vitro and in living cells, we show that LLPS of CGs is the main driver for cargo sorting at the TGN in specialized secretory cells. While it remains a challenge as to whether the tagged proteins in vitro behave similarly to that in the TGN lumen, we see that the conditions regulating LLPS of CGs in vitro also regulate the distribution of SG-targeted cargo at the TGN in living cells.

### In CG-LLPS, pH is the main driver while calcium is dispensable

In endocrine cells, the sorting process has been shown to strictly depend on the acidification of intracellular compartments because the addition of acidotropic drugs diverts regulated secretory proteins to the constitutive pathway, completely suppressing the formation of new SGs (Burgess and Kelly, 1987). CGs are synthesized in the ER and transported to the Golgi apparatus, where they are sorted and targeted to the SGs. Since LLPS of CGs facilitates cargo sorting toward the SGs, CGs must be in soluble form within the ER lumen and undergo LLPS at the TGN.

As proteins move along the secretory pathway, there is a pH gradient from an almost neutral pH (7.3) in the ER lumen that progressively becomes acidic from the cis- to the trans-Golgi, with a TGN pH of around 6.1 (Griffiths and Simons, 1986; Mellman et al., 1986; Anderson and Orci, 1988; Tooze and Tooze, 1986). Our results demonstrate that mildly acidic pH (6.1), resembling the TGN milieu, is a critical driver of LLPS, whereas calcium is not necessary; this contrasts with previous studies that have indicated calcium is required for the aggregation of CGs. These results are further strengthened by our findings on the behavior of the CGB_5(ED)/A mutant protein, which underwent LLPS on its own at high protein concentrations but failed to do so at low protein concentrations in the presence of calcium. Notably, these results also identify the five acidic stretches in CGB as potential calcium-binding sites.

Similarly, LLPS of CGA in the presence of crowding agents was favored at pH 6.1, and no aggregation of CGA was observed in the presence of 40 mM calcium. These results further emphasize that acidic pH and not calcium is the most critical parameter governing the LLPS of CGs. It is not surprising considering that calcium concentrations in the ER (400 µM; Pizzo et al., 2011) are considerably higher than those at the TGN. Since it is difficult to compute protein concentrations in the TGN and faithfully mimic the TGN environment in vitro, it cannot be completely ruled out that calcium may boost the inherent ability of CGB to undergo LLPS in vivo. We assume that protonation of some negatively charged amino acid residues of CGA (PI 4.58) or CGB (PI 5.03) at low pH conditions can promote LLPS by reducing electrostatic repulsion; consequently, the acidic milieu could induce the condensation of CGs in the TGN.

### Significance of the material properties of CGB for its function

Material properties of LLPS condensates play a significant role in regulating their function, and they are not necessarily linked with their LLPS capacity. In this context, RNA molecules (clients of LAF-1-IDRs) modulate LAF-1 viscosity inside the condensate. This change of material properties regulates the spatial partitioning of the different components during P-granule biogenesis (Elbaum-Garfinkle et al., 2015). Our experiments suggest that the regulation of the material state of CGB could play a significant role in this context. We base this hypothesis on the fact that the material state of CGB determines if it recruits clients. High concentrations of zinc induce the aggregation of CGB (Fig. 1 F). These aggregates do not bind to clients showing that liquid-like condensates are required for the reaction (Fig. 5, A and B). The abundance of zinc within the secretory pathway is spatially tightly controlled. CGB would only be exposed to high zinc concentrations in SGs as zinc transporters are highly abundant in their membrane (Lemaire et al., 2009; Nicolson et al., 2009). Based on these results, we suggest that CGB transitions from a liquid-like condensate to solid state as it moves along the secretory pathway from the TGN to the SGs, liquid in the TGN and solid in the granule. The function of CGB aggregation could be linked to its dissociation from clients in the granule. Interestingly, a recent preprint from the Ten Hagen laboratory has demonstrated cargo segregation within SGs from the fly salivary gland in a calcium- and pH-dependent fashion that also relies on glycosylation (Syed et al., 2021 *Preprint*). If our hypothesis holds true, it needs to be further tested in the future.

### LLPS drives protein sequence independent sorting at the TGN: A universal mechanism for receptor-independent sorting

According to the second tenet of the “sorting for entry” model, aggregation of CGs is thought to be a driving force at the TGN to mediate cargo sorting. Cargo molecules destined for SGs, such as proinsulin, neuropeptide Y (NPY), and processing enzymes PC1/3, PC2, and CPE, would be brought into the aggregate. In contrast, lysosomal hydrolases and other constitutive cargo molecules would be segregated from the aggregate, thus facilitating sorting. It remains unclear how molecules such as proinsulin are brought into the aggregate when there is no known receptor for this process and whether there is a specific sorting mechanism.

Our results challenge the specific sorting aspect of the “sorting by entry” model, as we show that ectopic expression of soluble proteins, such as LyzC-GFP or EqSol-GFP, in INS1 832/13 cells are secreted constitutively in HeLa cells, resulting in their targeting to SGs. Interestingly, overexpression of CatD, a lysosomal hydrolase, also showed the same phenotype. Thus, we propose that phase-separating granin proteins form scaffolds that act like a sponge that brings soluble proteins together into the condensed phase. Secretion of LyzC-GFP in response to glucose stimulation in INS1 832/13 cells suggests that LyzC and insulin remain in SGs and are released together in response to glucose stimulation. These findings suggest that proteins that are mistargeted to the SGs are not always retrieved by the “sorting by retention” model (Kuliawat and Arvan, 1994) which proposes that non-secretory proteins are removed by vesicles that bud off from the SG, thus mediating differential sorting of regulated versus constitutively secreted cargo.

Given our data showing segregation of proinsulin, M6PR, and IGF2R within the TGN volume and routing of lysosomal hydrolases to SGs upon overexpression, we suggest that at endogenous levels, lysosomal hydrolases are trafficked to lysosomes in a cargo receptor-dependent manner. Upon overexpression, the receptors are likely to be saturated, and the hydrolases become clients of the CG scaffold. The expression of granin proteins drives the targeting of soluble proteins to SGs as the dominant pathway. In parallel, the significance of CGs in targeting clients to SGs is highlighted by the fact that expression of CGA reroutes LyzC to ectopic SGs in HeLa cells. This is in line with previous findings showing that expression of *Xenopus laevis* CGA proteins in Cos7 cells directs co-expressed NPY to the ectopic CGA-derived granules (Montero-Hadjadje et al., 2009). Similarly, overexpression of CGB in AtT-20 cells mediates regulated secretion of Adrenocorticotrophin (ACTH; Natori and Huttner, 1996). NPY and ACTH, however, are targeted to SGs in specialized cells. Our results expand on this idea and show that CGA expression indeed reorganizes TGN sorting, as it can reroute LyzC, Eq-Sol to SGs, which are otherwise secreted constitutively from HeLa cells.

The nonspecific recruitment of clients to cells agreed with our in vitro assay findings. CGB scaffolds recruited LyzC and CatD. Our results refute the clear segregation of cargoes at the TGN based on a conserved sequence feature in the client proteins but instead suggest that weak interactions with the CG proteins result in recruitment to the condensates as they are formed in the TGN lumen. Importantly, the relative abundance of client proteins at the TGN rather than specific interactions potentially determines recruitment to the condensates and cargo sorting toward SGs. While the clients we tested were recruited to the condensates in vitro or routed to SGs in cells, antibody molecules when expressed in specialized secretory cells have been shown to be secreted through constitutive pathway (Rosa et al., 1989) or do not co-aggregate with SCG II in vitro (Gerdes et al., 1989). This could be a property of the antibody and the mechanism underlying this segregation must be investigated in the future. Recent work has demonstrated that client proteins are recruited to condensate of unfolded globular proteins through the principles of physical chemistry governed by complimentary interactions between the drivers and clients (Ruff et al., 2021). Similar studies would be necessary to determine what properties govern recruitment of CGB clients to condensates.

SGs are not limited to pancreatic β-cells but are also present in the α cells of the pancreas, which secrete glucagon as well as in specialized secretory cells of the adrenal and pituitary glands. We propose that instead of having unique receptors for different cargo molecules in these different cell types, cells utilize CG-driven LLPS as a sorting mechanism wherein cargo molecules are recruited to the granin sponge resulting in their targeting to SGs. Supporting this, the expression of GFP fused to a signal sequence directing it into the secretory pathway leads to its targeting to SGs in AtT-20 and PC-12 cells (El Meskini et al., 2001). Since molecules like insulin or NPY are crucial for physiology and metabolism, specialized cells like pancreatic β-cells express different members of the granin family, including CGA, CGB, SCG II, and VGF. Our studies have demonstrated that both CGA and CGB can undergo LLPS, although with a difference in capacity, and in silico analysis of VGF also identified disordered regions within the protein that may be indicative of LLPS. The capability of VGF to undergo LLPS needs to be tested, as ectopic expression of VGF also induces ectopic granule-like structures in NIH3T3 cells (Fargali et al., 2014). Even in the CGA/CGB dKO cells which we generated, we do not fully abolish stimulated insulin secretion, which could be due to the presence and compensation by VGF and SCG II in these cells.

Along similar lines, Spiess and colleagues showed that peptide hormone precursors including pro-vasopressin, pro-oxytocin, and POMC can form SGs in cell lines that normally lack regulated secretion (Beuret et al., 2004). POMC, for instance, contains a highly disordered region, and it can be speculated that it forms similar scaffolds in the TGN that promote sorting of SG-targeted proteins. This suggests that the abundance of a phase-separating scaffold protein in the TGN may regulate SG biosynthesis, which must be tested in future experiments.

While our studies explain how CGB scaffolds recruit clients for sorting to SGs, it remains unclear how the condensate is associated with the membrane for budding of the vesicles from the TGN. Previous research has suggested that CGs bind to the luminal leaflet of the TGN membrane, directly impacting membrane topology (Thiele et al., 1997; Tooze et al., 2001). Membrane-associated tethering proteins (either transmembrane proteins or peripheral membrane proteins) have been proposed to establish this connection (Dikeakos and Reudelhuber, 2007). Former work has proposed that soluble SCG III or CPE can act as a tether for CGA aggregates by binding to cholesterol (Hosaka et al., 2002). Based on our findings, it is conceivable that granin scaffolds also accumulate tethering proteins such as SCG III, thereby attaching the condensate to cholesterol-rich TGN membranes. Similarly, CGB has been shown to be associated with membranes which is dependent on the disulfide-bonded loop in the protein (Pimplikar and Huttner, 1992; Glombik et al., 1999). It remains unclear how the disulfide-bonded loop in CGB promotes membrane association. Alternatively, phase separation of condensed protein complexes has been proposed to cause membrane budding and fission (Alberti et al., 2019; Day et al., 2021). In this context, it was shown that the assembly of IDPs artificially targeted to a membrane surface induces membrane curvature due to a substantial compressive effect in the membrane plane (Day et al., 2021; Scheve et al., 2013; Busch et al., 2015; Snead et al., 2017). It would indeed be interesting to test whether CG condensates can drive membrane budding using an artificial membrane and if tethering to the membrane occurs by cholesterol binding.

Overall, our findings identify LLPS as an essential cellular process that is responsible for cargo sorting of soluble proteins toward SGs and that failure of LLPS impairs SG biogenesis.

## Materials and methods

### Cloning and constructs

For cloning, all the PCR amplifications were done using Phusion High-Fidelity DNA polymerase, ligations using T4 DNA ligase which were both obtained from Thermo Fischer Scientific. Final vectors were also generated using Gibson assembly (New England Biolabs; [NEB]) and/or using Gateway assembly (Invitrogen) as and when indicated. All the restriction digestion was carried out utilizing restriction enzymes from NEB. All the reactions were carried out using manufacturer’s protocols. Sequencing of the plasmids was carried out at Keck sequencing facility (Yale University).

For cloning of sfGFP and 6X-His tagged constructs of human CGA (pBT-PAF-CGA_sfGFP_6XHis) and CGB (pBT-PAF-CGB_sfGFP_6XHis), coding regions of CGA and CGB were cloned by PCR amplifications from expression plasmids from Origene (cat no. RC RC200492; CGA and cat no. RC201744; CGB). Super-folder GFP (sfGFP) was cloned by PCR amplification from the RINS1 construct (Schifferer et al., 2017). The nucleotide sequence encoding for 6X-His was incorporated into the reverse primer for amplification of sfGFP and thus was after the sfGFP. pBT-PAF vector was linearized using NheI-HF and NotI-HF restriction enzymes (NEB). The two individual fragments encoding CGA/CGB and sfGFP were then inserted into the digested vector using Gibson assembly. For generation of mEGFP tagged version of CGA, mEGFP was amplified from a plasmid expressing mEGFP tagged GM130, which was a kind of gift from the Rothman lab (Yale University, New Haven, CT).

For generating truncation mutants of CGB (pBT-PAF-CGB_N term-sfGFP_6XHis, pBT-PAF-CGB_C term-sfGFP_6XHis and pBT-PAF-CGB_C term-mCherry_6XHis), the N terminus of CGB, encoding amino acids 1–334 of CGB, was cloned by PCR amplification from pBT-PAF-CGB_sfGFP_6XHis construct and the C terminus, compromising signal sequence and 335-667 of CGB, was ordered as a gBlock gene fragment from IDT. 6XHis-tagged sfGFP and mCherry were amplified from the RINS1 construct. Final vectors were generated using Gibson assembly to insert CGB_N/CGB_C and sfGFP/mCherry into the digested pBT-PAF vector.

For generating pBT-PAF-CGB_5(ED)/A_sfGFP_6XHis, a gBlock gene fragment was ordered from IDT where all the acidic residues (Glutamic acid and Aspartic acid) in the five stretches of CGB were mutated to Alanine. This gene fragment and 6XHis-tagged sfGFP amplified from the RINS1 construct were inserted into the digested pBT-PAF vector to generate the final construct using Gibson assembly.

To generate pBT-PAF-6XHis_sfGFP, pBT-PAF-6XHis_sfGFP_Cab45 (Hecht et al., 2020) was digested using AscI and NotI-HF restriction enzymes and was PCR amplified to introduce NheI and NotI sites. The fragment was double-digested and then ligated into linearized pBT-PAF vector to obtain pBT-PAF_6XHis_sfGFP.

pLenti-LyzC_EGFP and pLenti-CatD_sfGFP were generated using gateway cloning. LyzC_EGFP was PCR amplified from pLPCX-LyzC_EGFP (Hecht et al., 2020) by the addition of appropriate nucleotides for gateway cloning reactions and cloned into the entry vector pDONR221 using the BP clonase reaction. LyzC_EGFP was cloned from the entry clone into the destination vector pLenti using LR clonase reaction to generate pLenti-LyzC_EGFP.

To generate pLenti-CatD_sfGFP, CatD fragment was PCR amplified from pBT-PAF-ssSUMO_CatD, by incorporating signal sequence from the CatD protein at the N-terminus and fused with sfGFP using extension overlap PCR also incorporating nucleotides for gateway reactions and the fragment was then cloned through sequential BP clonase and LR clonase reactions to obtain the destination vector.

To generate Halo-RUSH-CGB construct, DNA fragments for the signal peptide (SP) and streptavidin binding peptide (SBP) are a generous gift from Juan S. Bonifacino (National Institute of Child Health and Human Development, National Institutes of Health, Bethesda, MD). The piggyback SP-SBP-Halo-APP-mNeon backbone (the original backbone was a kind gift from Michael Ward, National Institute of Neurological Disorders and Stroke, National Institutes of Health, Bethesda, MD) was digested using XhoI/MluI and the CGB fragment was amplified by PCR from the RUSH-CGB-mcherry plasmid generated in the Von Blume laboratory. The CGB fragment was then subcloned into the C-terminus of SBP-Halo in place of APP-mNeon by Gibson assembly to generate the piggyback SP-SBP-Halo-CGB construct.

Strep-KDEL, used to generate the HeLa Strep-KDEL stable cell, was PCR amplified from Strep-KDEL_SBP-mCherry-GPI (plasmid #65295; Addgene) and assembled to a BamHI/PsrI digested TtTMPV-Neo viral backbone (plasmid #27993; Addgene). The neomycin resistance was afterward replaced with a puromycin encoding sequence.

Generation of recombinant adenovirus expressing CgB-CLIP has been previously described (Rohli et al., 2021 *Preprint*).

Sequences of primers used in the study are listed in Table 1 below.

**Table 1.** Sequences of primers used in this stud***y***

Primer name	Sequence
CGA_sfGFP_6XHis F1 FP	5′-GGC​GGC​CAT​CAC​AAG​TTT​GTA​CAG​CTA​GCA​TGC​GCT​CCG​CCG​CTG​TCC-3′
CGA_sfGFP_6XHis F1 RP	3′-CCG​GTG​GCG​ACC​GGT​GGA​TCC​AAG​CCC​CGC​CGT​AGT​GCC​TGC-5′
CGA_sfGFP_6XHis 2 FP	5′-GCA​GGC​ACT​ACG​GCG​GGG​CTT​GGA​TCC​ACC​GGT​CGC​CAC​CGG-3′
CGA_sfGFP_6XHis F2 RP	3′-CCA​GCA​CAC​TGG​ATC​AGT​TAT​CTA​TGC​GGC​CGC​TCA​GTG​ATG​ATG​ATG​GTG​ATG​GCT​GCC​CTT​GTA​CAG​CTC​GTC​CAT​GCC-5′
CGB_sfGFP_6XHis F1 FP	5′-GGC​GGC​CAT​CAC​AAG​TTT​GTA​CAG​CTA​GCA​TGC​AGC​CAA​CGC​TGC​TTC​TCA​GCC​TC-3′
CGB_sfGFP_6XHis F1 RP	3′-CAC​CGG​TGG​CGA​CCG​GTG​GAT​CCA​AGC​CCC​TTT​GGC​TGA​ATT​TCT​CAG​CTA​TCT​TCT​GTA​GTT​CC-5′
CGB_sfGFP_6XHis F2 FP	5′-GGA​ACT​ACA​GAA​GAT​AGC​TGA​GAA​ATT​CAG​CCA​AAG​GGG​CTT​GGA​TCC​ACC​GGT​CGC​CAC​CGG-3′
CGB_sfGFP_6XHis F2 RP	3′-CCA​GCA​CAC​TGG​ATC​AGT​TAT​CTA​TGC​GGC​CGC​TCA​TTA​GCT​GCC​CTT​GTA​CAG​CTC​GTC​CAT​GCC-5′
CGA_mEGFP_6XHis F1 FP	5′-GGC​GGC​CAT​CAC​AAG​TTT​GTA​CAG​CTA​GCA​TGC​GCT​CCG​CCG​CTG​TCC-3′
CGA_mEGFP_6XHis F1 RP	3′-AAC​AGC​TCC​TCG​CCC​TTG​CTC​ACC​ATA​GCT​CCT​GCA​CCG​GTG​GCG​ACC-5′
CGA_mEGFP_6XHis F2 FP	5′-GGT​CGC​CAC​CGG​TGC​AGG​AGC​TAT​GGT​GAG​CAA​GGG​CGA​GGA​GCT​GTT-3′
CGA_mEGFP_6XHis F2 RP	3′-CCA​GCA​CAC​TGG​ATC​AGT​TAT​CTA​TGC​GGC​CGC​TCA​GTG​ATG​ATG​ATG​GTG​ATG​CTT​GTA​CAG​CTC​GTC​CAT​GCC​GAG​AGT​GAT​CCC-5′
CGB_5(ED)/A_sfGFP_6XHis F1 FP	5′-GGC​GGC​CAT​CAC​AAG​TTT​GTA​CAG​CTA​GCA​TG-3′
CGB_5(ED)/A_sfGFP_6XHis F1 RP	3′-CAC​CGG​TGG​CGA​CCG​GTG​GAT​C-5′
CGB_5(ED)/A_sfGFP_6XHis F2 FP	5′-GCT​TCA​GAA​AAT​AGC​GGA​GAA​GTT​CTC​ACA​ACG​AGG​CTT​GGA​TCC​ACC​GGT​CGC​CAC​CGG-3′
CGB_5(ED)/A_sfGFP_6XHis F2 RP	3′-CCA​GCA​CAC​TGG​ATC​AGT​TAT​CTA​TGC​GGC​CGC​TCA​GTG​ATG​ATG​ATG​GTG​ATG​GCT​GCC​CTT​GTA​CAG​CTC​GTC​CAT​GCC-5′
CGB_N term_sfGFP_6XHis F1 FP	5′-GGC​GGC​CAT​CAC​AAG​TTT​GTA​CAG​CTA​GCA​TGC​AGC​CAA​CGC​TGC​TTC​TCA​GCC​TC-3′
CGB_N term_sfGFP_6XHis F1 RP	3′-CAC​CGG​TGG​CGA​CCG​GTG​GAT​CCA​AAG​CCC​TGT​AGT​GGG​TTG​AAT​GGT​GGT​CCC-5′
CGB_N term_sfGFP_6XHis F2 FP	5′-GGG​ACC​ACC​ATT​CAA​CCC​ACT​ACA​GGG​CTT​TGG​ATC​CAC​CGG​TCG​CCA​CCG​G-3′
CGB_N term_sfGFP_6XHis F2 RP	3′-CCA​GCA​CAC​TGG​ATC​AGT​TAT​CTA​TGC​GGC​CGC​TCA​GTG​ATG​ATG​ATG​GTG​ATG​GCT​GCC​CTT​GTA​CAG​CTC​GTC​CAT​GCC-5′
CGB_C term_sfGFP_6XHis F1 FP	5′-GGC​GGC​CAT​CAC​AAG​TTT​GTA​CAG​CTA​GCA​TG-3′
CGB_C term_sfGFP_6XHis F1 RP	3′-CAC​CGG​TGG​CGA​CCG​GTG​GAT​C-5′
CGB_C term_sfGFP_6XHis F2 FP	5′-GGA​ACT​ACA​GAA​GAT​AGC​TGA​GAA​ATT​CAG​CCA​AAG​GGG​CTT​GGA​TCC​ACC​GGT​CGC​CAC​CGG​TG-3′
CGB_C term_sfGFP_6XHis F2 RP	3′-CCA​GCA​CAC​TGG​ATC​AGT​TAT​CTA​TGC​GGC​CGC​TCA​GTG​ATG​ATG​ATG​GTG​ATG​GCT​GCC​CTT​GTA​CAG​CTC​GTC​CAT​GCC-5′
CGB_C term_mCherry_6XHis F1 FP	5′-GGC​GGC​CAT​CAC​AAG​TTT​GTA​CAG​CTA​GCA​TG-3′
CGB_C term_mCherry_6XHis F1 RP	3′-CAC​CGG​TGG​CGA​CCG​GTG​GAT​C-5′
CGB_C term_mCherry_6XHis F2 FP	5′-TTG​GAT​CCA​CCG​GTC​GCC​ACC​GGT​GCA​GGA​GCT​ATG​GTG​AGC​AAG​GGC​GAG​GAG​GAT​AAC​ATG​G-3′
CGB_C term_mCherry_6XHis F2 RP	3′-CCA​GCA​CAC​TGG​ATC​AGT​TAT​CTA​TGC​GGC​CGC​TCA​GTG​ATG​ATG​ATG​GTG​ATG​CTT​GTA​CAG​CTC​GTC​CAT​GCC​GCC​G-5′
pLenti_LyzC_EGFP_attB_FP	5′-GGG​GAC​AAG​TTT​GTA​CAA​AAA​AGC​AGG​CTA​TGA​AGG​CTC​TCA​TTG​TTC​TGG​GGC​TTG​TCC​TCC-3′
pLenti_LyzC_EGFP_attB_RP	3′-GGG​GAC​CAC​TTT​GTA​CAA​GAA​AGC​TGG​GTT​TAC​TTG​TAC​AGC​TCG​TCC​ATG​CCG​AGA​GTG​ATC​CCG-5′
pLenti_CatD_sfGFP_F1_FP	5′-GGG​GAC​AAG​TTT​GTA​CAA​AAA​AGC​AGG​CTA​TGC​AGC​CCT​CCA​GCC​TTC​TGC​CGC​TCG​CCC​TCT​GCC​TGC​TGG​CTG​CAC​CCG​CCT​CCG​CGC​TCG​TCA​GGA​TCC​CGC​TGC​ACA​AGT​TCA-3′
pLenti_CatD_sfGFP_F1_RP	3′-ACC​GGT​GGC​GAC​CGG​TGG​ATC​CAA​GAG​GCG​GGC​AGC​CTC​GGC​GA-5′
pLenti_CatD_sfGFP_F2_FP	5′-TCG​CCG​AGG​CTG​CCC​GCC​TCT​TGG​ATC​CAC​CGG​TCG​CCA​CCG​GT-3′
pLenti_CatD_sfGFP_F2_RP	3′-GGG​GAC​CAC​TTT​GTA​CAA​GAA​AGC​TGG​GTT​TAG​CTG​CCC​TTG​TAC​AGC​TCG​TCC-5′
SP_SBP_Halo_CGB_FP	5′-CGC​GCG​CTG​GCT​GTC​CAC​GCT​CGA​GAT​TTC​CGG​CGG​CGG​CAG​CAT​GCC​AGT​GGA​TAA​CAG​GAA​CCA​C-3′
SP_SBP_Halo_CGB_RP	3′-GCA​GCC​TGC​ACC​TGA​GGA​GTG​AAT​TCA​TTA​GCC​CCT​TTG​GCT​GAA​TTT​CTC-5′

Sequences of guide RNAs used in the study are listed in Table 2 below.

**Table 2.** Sequences of guide RNAs

Target gene	gRNA	Target sequence
Chga	gRNA1	5′-GCA​TAG​CGA​GCC​GGA​CGG​TG-3′
	gRNA2	5′-TGT​GCG​CCG​GGC​AAG​GTG​AG-3′
	gRNA3	5′-GCG​AGT​CGG​AGA​TGA​CCT​CC-3′
	gRNA4	5′-GTT​GTC​TGC​ATG​AGG​CCA​CG-3′
Chgb	gRNA1	5′-TGG​CGA​GCG​GCA​CCA​TGC​AG-3′
	gRNA2	5′-AAA​CCC​GGT​ACT​TGC​AGC​GC-3′
	gRNA3	5′-TCA​GGA​CTT​GCC​GGC​ACT​CA-3′
	gRNA4	5′-TCA​CTA​GAG​GCT​CGA​ACA​TG-3′

### Cell culture

HEK293 and HeLa cells were maintained in DMEM high glucose (GIBCO) supplemented with 10% fetal bovine serum (FBS; GIBCO), 100 U/ml penicillin, and 100 µg/ml streptomycin in 5% carbon dioxide at 37°C. Rat insulinoma INS1 832/13 cells and INS1 832/13 cells expressing SNAP tagged proinsulin or CatD-GFP and INS1 832/13 cells dually expressing SNAP-tagged proinsulin and LyzC-GFP were maintained in RPMI 1640 (cat no. 11879-020; GIBCO) containing 11 mM glucose and supplemented with 10% FBS, 100 U/ml penicillin and 100 µg/ml streptomycin, 10 mmol/l HEPES (americanBIO), 2 mmol/l Glutamax (GIBCO), 1 mmol/l sodium pyruvate (GIBCO) and 50 µmol/l β-mercaptoethanol (americanBIO) in 5% carbon dioxide at 37°C.

### Generation of stable cell lines for protein expression

Doxycycline inducible HEK293 stably expressing GFP, and His-tagged proteins or mCherry and FLAG tagged proinsulin proteins were generated using the transposon-based piggyBac system (Li et al., 2013). HEK293 cells were transfected with a mixture of pBT-PAF, PB-RN and PBase (8:1:1 ratio) using Lipofectamine 2000 (Invitrogen) according to manufacturer’s protocol. Medium was exchanged after 4–6 h. 48-h post-transfection cells stably expressing the plasmid cocktail were selected by treatment with the antibiotics puromycin dihydrochloride (10 µg/ml; Sigma-Aldrich) and G418 disulfate salt (500 µg/ml; Sigma-Aldrich). Stable lines were then frozen down for subsequent use.

### Protein expression, purification of His-tagged proteins

GFP/mCherry- and His-tagged proteins were purified from culture supernatants as described in Fig. 2 B using nickel-based column chromatography. Doxycycline-inducible HEK293 stably expressing GFP/mCherry- and His-tagged proteins were expanded in 10–20 15-cm dishes. Fully confluent cells were induced for protein expression by the addition of doxycycline monohydrate (1 µg/ml; LKT Laboratories) in serum-free DMEM high glucose also containing proteinase inhibitor aprotinin (1 µg/ml; Sigma-Aldrich) and antibiotic A23187 (1 µg/ml; Thermo Fisher Scientific Alfa Aesar) for 15–20 h. Medium was collected and pre-cleared of cells by centrifugation and then by filtration using a 0.45 µm filter. The supernatant was then loaded onto a Ni-NTA column generated from cOmplete His-tag Purification Resin (Roche), which was equilibrated in sodium phosphate buffer, containing 500 mM NaCl; pH 6.8. The column was washed with the equilibrating buffer containing 10 mM imidazole and then eluted using the equilibrating buffer containing 250 mM imidazole. Imidazole was removed from the proteins by buffer exchanging the protein with Tri-HCl buffer containing 500 mM NaCl; pH 6.8 as well as 10% (vol/vol) glycerol and stored in it at −80°C after snap-freezing the protein in batches in liquid nitrogen.

### Lentiviral production

Lentiviruses were generated using HEK293 cells. HEK293 cells were plated on poly-Lysine-coated 10 cm^2^ dishes and grown in DMEM high glucose medium. The following day they were transfected with a plasmid cocktail containing packaging plasmid containing the gene of interest along with psPAX2 (Gag, Pol, Rev, and Tat), pMD2.G (VSV-G) using Lipofectamine 2000 according to manufacturer’s protocol. On the next day, the medium was changed to INS1 832/13 culture medium since the viruses were to be used for infecting INS1 832/13. 48-h post-transfection medium containing virus particles was collected from cells and passed through a 0.45 µm filter and stored at 4°C. HEK293 cells were again supplemented with INS1 832/13 culture medium for one more round of collection. 72-h post-transfection collection protocol was repeated and collection from 2 d was pooled and either used immediately for infection or stored at −80°C after aliquoting in batches.

### Generation of INS1 832/13 stable cell lines

Lentiviruses were used for infection of INS1 832/13 cells for generation of stable cell lines expressing LyzC-EGFP and CatD-sfGFP. Stable cell lines were selected after antibiotic selection.

### Generation of HeLa Strep-KDEL cell lines

Retroviral particles containing the Strep-KDEL plasmid were used to stably infect HeLa cells. Cells were selected with puromycin (1 mg/ml; A1113803; Gibco) and a single-cell clone was isolated. The HeLa Strep-KDEL stable cell line was grown in DMEM high glucose (D6429; Sigma-Aldrich) supplemented with 10% fetal bovine serum (FBS; F7424; Sigma-Aldrich) and MycoZap Plus-CL (VZA-2012; Lonza) and was kept at 37°C in a humidified 5% CO_2_ atmosphere.

### In vitro droplet formation assay

Droplet formation of CGB-GFP without any crowding agents was monitored by microscopy after exchanging the storage buffer in which proteins were stored, containing high salt, to phase separation buffer, i.e., Tris-HCl; pH 6.1 containing 150 mM NaCl and 2.5% glycerol. 10 μl solution was plated on a coverslip glass bottom imaging dishes (Cellvis) and the droplets which were settled on the coverslip were imaged using Zeiss 880 confocal microscope by excitation using the 488 nm laser and imaging using a 63×/1.4 oil objective at room temperature.

For inducing droplet/aggregate formation in the presence of divalent cations, CGB solution was centrifuged at 4°C on a benchtop Sorvall Legend Micro 21R centrifuge at a maximum speed for 20 min to pre-clear of any existing droplets. Droplet formation was then induced by mixing 2.5 µM CGB-GFP (final concentration) with different concentrations of divalent cations in the phase separation buffer, followed by imaging of droplets which have been settled to the bottom of a coverslip glass bottom imaging dish.

For inducing CGB-GFP droplets in the presence of crowding agents, 2.5 µM CGB-GFP (final concentration) was mixed with 1–3% PEG 8000 (Thermo Fischer scientific) and imaged on Zeiss 880 confocal microscope with a 63×/1.4 oil objective at room temperature. Similarly, CGA-GFP droplets were induced using 3–5% PEG 8000 or in the presence of 5% Dex500 (Sigma-Aldrich).

### Fluorescence recovery after photobleaching and analysis

Partial FRAP experiments were carried out on settled droplets within 20–30 min of initiation of the experiment. FRAP module on the Zeiss 880 confocal microscope with a 63×/1.4 oil objective was used for the experiment. Imaging was done at room temperature. In all the experiments other than those described in Fig. 3 D, bleaching and subsequent imaging was performed using the excitation wavelength of 488 nm. A circular region of interest within the droplet was used for photobleaching with the laser operating at 100% capacity, and recovery of fluorescence within the bleached region was monitored using continuous imaging for next 1–1.5 min with the laser power <1% of its full capacity. For the experiment described in Fig. 3 D, a combination of 488 and 405 nm lasers was used for bleaching while recovery was monitored only by excitation at 488 nm. For analysis, the intensity profile in the bleached region was corrected using an unbleached region from other droplets in the image and the FRAP curves were then plotted using the corrected data sets.

### Effects of pH on droplet formation

To test the effects of pH on droplet formation of CGB-GFP, CGB-GFP protein in storage buffer was exchanged to either phase separation buffer i.e., Tris-HCl; pH 6.1 containing 150 mM NaCl and 2.5% glycerol or in Tris-HCl; pH 7.3 containing 150 mM NaCl and 2.5% glycerol using Amicon concentrator (30 kD; 3×) and droplet formation was monitored by plating the protein solutions on a coverslip glass-bottom imaging dish, followed by imaging on Zeiss 880 confocal microscope with a 63×/1.4 oil objective at room temperature.

To test the effects of pH on droplet formation of CGA-GFP, CGA-GFP protein in storage buffer was exchanged similarly to either phase separation buffer i.e., Tris-HCl; pH 6.1 containing 150 mM NaCl and 2.5% glycerol or in Tris-HCl; pH 7.3 containing 150 mM NaCl and 2.5% glycerol and droplet formation was induced in the presence of 5% Dex500 and monitored by microscopic imaging.

### Client partitioning assay

Client proteins were exchanged from their storage buffers into phase separation buffer. To monitor the recruitment of Cy3-tagged proinsulin (1336PN050; R&D systems), CGB-GFP (16 µM final concentration) and Cy3-proinsulin (1 µM final concentration) were mixed, and droplets were imaged using Zeiss 880 microscope in both GFP and the Cy3 channels using a 63×/1.4 oil objective at room temperature.

For monitoring the recruitment of Cy3-LyzC (LS1-S3-1; Nanocs) and Cy3-CatD (ab276483), CGB-GFP and the clients were mixed in 2.5:1 molar ratios and droplet formation was induced using either 20 mM calcium or 3% PEG 8000.

For quantifying recruitment of Cy3-LyzC to condensates or aggregates, imaging was performed under same settings in both GFP and the Cy3 channel. Region of interest was drawn using the GFP channel (CGB-GFP) in imageJ to measure the intensity in the GFP channel and in the Cy3 channel. Data were represented as ratio of signal in Cy3 channel to that in the GFP channel and compared in calcium-induced and zinc-induced conditions.

### Live cell imaging

For imaging of RUSH assays 0.09 × 10^6^ HeLa Strep-KDEL cells were plated onto matrigel-coated glass coverslips (CB00250RAC; Menzel-Gläser). FuGENE 6 was used to transfect constructs encoding SP-SBP-Halo-CGB in combination with Golgi-mEGFP (GAL-T, plasmid #182877; Addgene) or LyzC-GFP the following day. 2 h after transfection, media was replaced with fresh complete DMEM. The next day, cells were incubated for 1 h at 37°C with fresh complete DMEM containing 646 HALO Dye (200 nM; GA112A; Promega), washed twice with PBS 1X and imaged in an Elyra 7 with Lattice SIM^2^ microscope (Zeiss) equipped with an environmental chamber (temperature controlled at 37°C, humidified 5% CO_2_ atmosphere), two PCO.edge sCMOS version 4.2 (CL HS) cameras (PCO), solid state diode continuous wave lasers and a Zeiss Plan-Apochromat 63×/1.4 Oil DIC M27, all under the control of ZEN black software (Zeiss). D-biotin (B4501; Sigma-Aldrich) at a final concentration of 500 µM was added to start the RUSH.

For imaging the dynamics of proinsulin puncta, INS1 832/13 cells were transfected with RINS1 construct and imaged on the following day using 63×/1.4 oil objective on Zeiss 880 microscope equipped with an environmental chamber (temperature controlled at 37°C, humidified 5% CO_2_ atmosphere).

### Protein detection by immunoblotting, Coomassie staining, and immunofluorescence

For Western blotting, proteins were transferred from SDS gels to a nitrocellulose membrane using wet blot system from Bio-Rad laboratories. After transfer to membranes, they were incubated with 5% milk made in Tris-buffered saline containing 0.1% Tween-20 for at least 1 h. Membranes were incubated with specific primary (overnight incubation) and HRP-coupled secondary antibodies (1 h incubation) and proteins were detected using chemiluminescence (Thermo Fischer Scientific) using ChemiDoc imaging system (Bio-Rad laboratories).

For detecting protein using Coomassie staining, SDS gels were rinsed with water following which stained using 0.1% Coomassie Brilliant Blue solution made in 40% vol/vol methanol and 10% vol/vol acetic acid and destained using 40% vol/vol methanol and 10% vol/vol acetic acid solution and stored in water prior to imaging.

For immunofluorescence-based detection of proteins within the TGN volume, INS1 832/13 cells were plated on HTB9-coated coverslips at low density and cultured overnight as previously described (Bearrows et al., 2019). Cells were fixed in 10% neutral-buffered formalin and incubated overnight with primary antibodies as indicated. Highly cross-adsorbed fluorescent conjugated secondary antibodies (whole IgG, donkey anti-guinea pig-AlexaFluor 488, donkey anti-rabbit rhodamine red-X, donkey anti-mouse AlexaFluor 647; Jackson ImmunoResearch) were used for detection. Cells were counterstained with DAPI and mounted using Fluorosave (Calbiochem).

For the detection of proteins post Brefeldin A, concamaycin A and ammonium chloride treatments, cells were fixed using 4% paraformaldehyde made in PHEM buffer and then permeabilized using PHEM buffer containing 0.3% NP-40 and 0.05% Triton X-100 for 5 min. Primary and secondary antibodies were diluted in PHEM buffer containing 0.05% NP-40, 0.05% Triton X-100 and 5% serum. Coverslips were mounted using Prolong Gold also containing DAPI (Thermo Fischer Scientific) and imaged using Zeiss 880 confocal microscope with a 63×/1.4 oil objective at room temperature.

For all other experiments, cells were either grown on coverslips or glass-bottom dishes (Cellvis) and fixed using 4% paraformaldehyde (Electron Microscopy Sciences) made in phosphate-buffered saline (PBS) and permeabilized using 0.4% saponin (Sigma-Aldrich) made in PBS containing 4% BSA (americanBIO) for at least 1 h. Cells were stained in respective primary antibodies and fluorophore conjugated secondary antibodies (Invitrogen). Coverslips were mounted using Prolong Gold also containing DAPI and imaged using Zeiss 880 confocal microscope with a 63×/1.4 oil objective at room temperature.

### Pulse chase experiments

For SNAP-tag and CLIP-tag labeling, INS1 832/13 cells stably expressing proCpepSNAP (Bearrows et al., 2019) were treated with recombinant adenovirus expressing CgB-CLIP and pulse-chase labeled as previously described (Rohli et al., 2021 *Preprint*) for the indicated times. For experiments involving concanamycin A, 100 nM concanamycin A was added in two washing steps prior to fixation.

### 3D rendering of TGN volume

Images of INS1 832/13 stained with antibodies to TGN38 and soluble cargo or receptors were captured on a Leica SP8 confocal microscope using a HC PL APO CS2 40×/1.4 oil objective with 5× zoom as z-stacks (5 per set, 0.3 μm step, 0.88 μm optical section) and deconvolved (Huygen’s Professional). Golgi volume mask was created using surface rendering of the Golgi identified by TGN38 immunostaining (Imaris, Bitplane) and line intensity profile of immunostaining within the Golgi was generated using ImageJ software.

For analysis of PC2 distribution in wild-type and dKO cells, TGN38 volume generated using Imaris 9 after deconvolution and was superimposed on the PC2 channel to create surfaces in the PC2 channel within the TGN38 volume.

### Electron microscopy

C57BLKS/J mice were purchased from Jackson Laboratories and maintained as an active breeding colony. Islets were isolated from 14-wk-old male mice through collagenase V digestion and purified using Histopaque 1077 and 1119. Islets were allowed to recover overnight in RPMI supplemented with 10% fetal bovine serum and 1% penicillin and streptomycin and maintained at 37°C in 5% CO_2_. All animal protocols were approved by the University of Iowa Institutional Animal Use and Care Committee. Isolated islets were washed by PBS and fixed in 2.5% glutaraldehyde and 4% paraformaldehyde at 4°C overnight. Islets were washed with 0.1 M sodium cacodylate three times and post-fixed in freshly made 1% (w/v) reduced OsO4 and 1.5% (w/v) cyanoferrate in 0.1 M sodium cacodylate for 1 h. Islets were rinsed three times with water and processed for successive dehydration (50% ethanol, 2 × 5 min; 70% ethanol, 2 × 5 min, 90% ethanol, 2 × 5 min; 100% ethanol, 3 × 15 min; and propylene oxide, 15 min). Infiltration was performed using Epon-Mix, and samples were incubated at 60°C overnight for polymerization as previously described (Joshi et al., 2014; Tang et al., 2010). Resin blocks were cut to ultrathin (50–70 nm) sections with a diamond knife and mounted on Formvar-coated copper grids. Grids were double-contrasted with 2% uranyl acetate and then with lead citrate at room temperature and washed immediately with excessive water. Images were captured at 8,000× and 12,000× magnifications by a JEOL JEM-1400 transmission electron microscope. All EM-related reagents were from Electron Microscopy Sciences (EMS).

For imaging of budding vesicles in pituitary glands, deeply anesthetized mice were flushed with a pre-warmed (37°C) calcium and magnesium-free buffer containing DPBS, 10 mM HEPES, 0.2 mM EGTA, 0.2% BSA, 5 mM glucose, and 10 mM KCl for 2 min followed by perfusion with fresh and pre-warmed fixative buffer (2.5% glutaraldehyde, 2% paraformaldehyde in 0.15 M cacodylate buffer) for 3 min using a peristaltic pump through the left ventricle (Pasqua et al., 2016). Pituitary gland was put in fixative overnight (2 h at room temperature and 12 h at 4°C), post-fixed in 1% osmium tetroxide in 0.1 M sodium cacodylate buffer for 1 h on ice, and stained en bloc with 2% uranyl acetate for 1 h on ice. The stained tissues were dehydrated in ethanol (20–100%) and embedded with Durcupan (Sigma-Aldrich). Ultrathin (50–60 nm) sections were post-stained with uranyl acetate and lead stain. Samples were viewed using a JEOL JEM1400-plus TEM (JEOL) and photographed using a Gatan OneView digital camera with 4k × 4k resolution.

### Imaging ectopic granules in HEK293 cells

HEK293 cells stably expressing CGB-GFP and truncation mutants of CGB were plated on glass-bottom imaging dishes from Cellvis. Cells were induced from protein expression by the addition of doxycycline monohydrate (1 µg/ml; LKT Laboratories) for 10 h following which cells were fixed using 4% paraformaldehyde (Electron Microscopy Sciences) and co-stained with DAPI to label the nuclei. Cells were imaged on the Zeiss 880 confocal microscope. The bottom plane closest to the coverslip was imaged using a 63×/1.4 oil objective at room temperature. In some cells expressing GFP-tagged C-terminal portion of CGB, we observed signal from the nucleus and these cells were excluded from imaging.

### Labeling of proinsulin and CatD

Recombinant human CatD protein was purchased from Abcam. CatD and proinsulin were labeled with Cy3 using a kit from AAT Bioquest (cat no. 1290) according to manufacturer’s protocols. Labeled proteins were buffer-exchanged using the phase separation buffer and used immediately.

### Secretion assays

Secretion of LyzC-EGFP in response to glucose stimulation was monitored from cells stably expressing LyzC-EGFP and SNAP-tagged proinsulin. Cells were plated in a 10 cm^2^ dish and assayed at >80% confluency. On the previous day of the assay, cells were incubated in INS1 832/13 containing medium but with 5 mM glucose concentration instead of the regular 11 mM glucose concentration. After overnight incubation, cells were incubated in secretion assay buffer as described in (Hohmeier et al., 2000) for 2 h followed by incubation in either serum-free INS1 832/13 medium with either 3 mM glucose (basal) or 15 mM glucose and 35 mM KCl (stimulated) for 2 h. Medium was collected after centrifugation at 800 *g* for 5 min for removal of any floating cells and then concentrated using Amicon concentrators (3 kD cut off). Levels of proteins in supernatants and cell lysates were analyzed using western blotting and probed using α-GFP antibody to detect the levels of LyzC-GFP and α-SNAP-tag antibody to monitor the secretion of SNAP tagged C-peptide, a proxy for insulin secretion. Intensity of bands in cell lysates and supernatants were measured using densitometry and amount of LyzC-GFP protein in the supernatant was normalized to the levels in cell lysates in both basal and stimulated conditions.

### Brefeldin A treatment

INS1 832/13 cells were plated on glass coverslips and treated with Brefeldin A (5 µg/ml; Cell Signaling Technology) for 3 h at 37°C. Control cells were treated with 0.05% DMSO.

### Antisera

Antibodies used for immunofluorescence were as follows: Insulin (MA1-10517; Thermo Fischer Scientific and A0564; Dako), CGB (259 10; Synaptic systems), CGA (NB120-15160; Novus biologicals), TGN 38 (NB300-575; Novus biologicals and 610898; BD Biosciences), CPE (13710-1-AP; Proteintech) M6PR (16795-1-AP; Proteintech), IGF2R (20253-1-AP; Proteintech), SCG III (10954-1-AP; Proteintech), PCSK2 (PA1-058; Invitrogen), HA (11867423001; Roche/Sigma-Aldrich), Cathepsin B (AF965; R&D systems).

For purposes of western blotting, antibodies against GFP (11814460001; Roche/Sigma-Aldrich) SNAP-tag (P9310S; NEB), and β-actin (A5441; Sigma-Aldrich) were used.

### Glucose-stimulated insulin secretion assay

INS1 832/13 cells were seeded into 6-well plates before experiments until they reach 80–90% confluency. 1 d before experiments, cell-culture media were changed to RPMI5 (normal growth media but with 5 mM glucose) and buffers HBSS3 (114 mM NaCl, 4.7 mM KCl, 1.2 mM KH_2_PO_4_, 1.16 mM MgSO_4_, 20 mM HEPES, 2.5 mM CaCl_2_, 25.5 mM NaHCO_3_, 0.2% BSA, 3 mM glucose, pH 7.2) and HBSS15 (same as HBSS3 but with 15 mM glucose) were prewarmed in a TC incubator as needed. On the experiment day, cells were washed with HBSS3 and then incubated with HBSS3 for 90 min in a TC incubator. After washing with HBSS3, cells were incubated with 1 ml of HBSS3 for 1 h followed by 1 h with 1 ml of HBSS15 as needed in a TC incubator. Supernatants were collected after each incubation and spun down at 1000 RPM, 4°C for 5 min to remove any loose cells by centrifuge (Beckman Coulter Centrifuge 5910R). Insulin release was determined by an insulin ELISA kit (ALPCO rat high range insulin ELISA, cat no. 80-INSRTH-01) and proinsulin release was determined by a proinsulin ELISA kit (Mercodia Rat/Mouse Proinsulin ELISA, cat no. 10-1232-01) according to the manufacturers’ instructions. The remaining cells were washed with ice-cold PBS and lysed with 1 ml of ice-cold 1% Triton. Cell lysates were used to measure total protein content with the Coomassie protein assay reagent (cat no.1856209; Thermo Fisher Scientific). Colorimetric measurements were carried out by SpectraMax iD3 Multi-Mode Microplate Reader. Insulin and proinsulin release were normalized to total protein content in cells and relative insulin release upon glucose stimulation was obtained as the fold change when increasing glucose concentration from 3 to 15 mM.

### Generation of double knockout cell lines by CRISPR/Cas9

CRISPR/Cas9 targets for rat gene Chga and Chgb were selected and designed based on the ChopChop algorithm to introduce double-stranded breaks and non-homologous end joinings into the genes. A pair of oligos for Chga target were cloned into lentiCRISPRv2 hygro and lentiCRISPRv2 blast (plasmid #98291 and #98293; Addgene), respectively, and a pair of oligos for Chgb target were cloned into LentiCRISPRv2-mCherry (plasmid #99154; Addgene) and lentiviruses were generated according to Zhang lab GeCKO website (http://genome-engineering.org/gecko/) information. INS1 832/13 cells were infected by lentiviruses conferring the gRNAs targeting Chga and selected with 400 μg/ml hygromycin and 10 μg/ml blasticidin sequentially. Positive knockout clones were confirmed by western blotting and qRT-PCR and expanded. INS1 832/13 Chga-KO cells were then infected by lentiviruses conferring both gRNAs targeting Chgb and single clones of mCherry-positive cells were picked out for further validation of positive knockout clones.

### cDNA preparation for qRT-PCR

Total RNA was extracted from cell lysates using RNeasy Mini Kit (Qiagen). 1 µg of RNA was used for reverse-transcription using RevertAid RT Reverse Transcription Kit (Thermo Fisher Scientific) to generate cDNA. The cDNA samples were stored at −80°C for qRT-PCR assays.

### qRT-PCR

Primer design for qRT-PCR experiments was based on the PerlPrimer algorithm. All qRT-PCR experiments were performed using 5 μl SYBR(R) green qPCR Readymix iQ (KiCqStart; Sigma-Aldrich), 1 μl cDNA (1:400 dilution), 1 μl primer mix (5 µM each for sense and antisense primers), and 3 μl nuclease-free water in triplicates. Amplification with the respective primers was performed and recorded with CFX96 Real-Time PCR System and data were further analyzed with CFX Maestro software (Bio-Rad).

### Image quantification

Fiji was used for analyzing images from in vitro phase separation assays. For each image, droplet particles with size larger than 0.01 μm^2^ were thresholded and selected. The selection was applied to the original images for measurement. For each image, the size of droplets was measured, and sum of droplet sizes was divided by the area of a field of view to obtain the area coverage of condensates. The distribution frequency of droplets or condensate coverage from a set of images was plotted using Prism 9.

Fiji and in-built measurement were used for analyzing images of ectopic granules in HEK293 cells. To quantify the area covered by granules in cell, each cell body outlined by peripheral GFP signals was captured as an ROI. For each ROI, GFP signals from cytosolic granules were measured and signals from the nucleus (stained with DAPI) or Golgi were excluded. Area covered by granules was divided by total area of an ROI and area coverage from a set of images was plotted using Prism 9.

### Statistics

Statistical analysis was performed using Prism 9. Normality of the distribution was tested using Shapiro-Wilk test. For normally distributed data, a *t* test was used while Mann–Whitney test was used for data which did not show normal distribution. Statistical test used and the P values are described in figure legends.

### Online supplemental material

Fig. S1 shows experiments related to Fig. 1 characterizing LLPS behavior of CGA-GFP at different protein concentrations in the presence of PEG 8000, calcium dependence, phase diagrams of CGB-GFP with calcium and effects of titrating zinc concentrations on higher order assemblies of CGB-GFP. Fig. S2 is related to Fig. 2 and shows control experiments depicting Golgi morphology upon concanamycin A and ammonium chloride treatment and constitutive secretion of proinsulin upon concanamycin A treatment. Fig. S3 is related to Fig. 3 showing colocalization of insulin with CGB, CGA, and SCG II at steady state. It also has control experiments validating knockdown of CGA and CGB in double knock out cells using immunofluorescence, western blots, and qPCR. Fig. S4 is related to Fig. 4. It shows colocalization of proinsulin with CPE within the TGN volume and its segregation from cation-dependent and independent mannose-6-phosphate receptors within the volume. There is also an experiment demonstrating segregation of proinsulin from Cathepsin B at cell endogenous levels. Fig. S5 is related to Fig. 6 and shows behavior of truncation mutants of CGB without addition of PEG 8000 and further controls demonstrating that GFP and mCherry-tagged C-terminal part of CGB behaves similarly. There is also a western blot to monitor the levels of CGB-GFP and the truncation mutants in HEK293 cells stably expressing the proteins upon induction using doxycycline. Videos 1 and 2 are related to Fig. 2 F and show the projection of the proinsulin signal at the TGN in control and upon concanamycin A treatment. Video 3 is related to Fig. 2 H which captures the dynamics of RUSH-CGB at the Golgi apparatus. Video 4 is related to Fig. 4 G demonstrating recruitment of LyzC-GFP to CGB condensates at the Golgi apparatus upon release of RUSH-CGB using biotin.

## Supplementary Material

SourceData F1 is the source file for Fig. 1.

SourceData F4 is the source file for Fig. 4.

SourceData FS1 is the source file for Fig. S1.

SourceData FS3 is the source file for Fig. S3.

SourceData FS5 is the source file for Fig. S5.
