# Supplementary figures and images for: Liquid–liquid phase separation facilitates the biogenesis of secretory storage granules
Source: J Cell Biol. 2022 Sep 29;221(12):e202206132. doi: 10.1083/jcb.202206132 (PMC9526250; doi:10.1083/jcb.202206132)

# Source Data F1

CGB-GFP

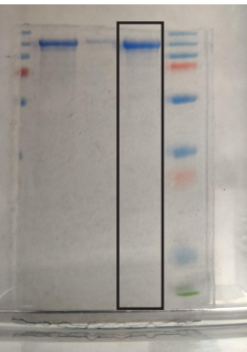

CGB\_(5ED)/A-GFP

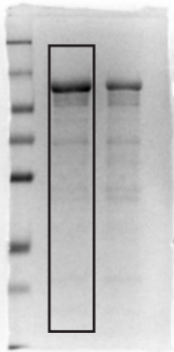

Supplement: SourceData F1 — is the source file for Fig. 1. [file JCB_202206132_SourceDataF1.pdf]

# Source Data FS1

CGA-GFP

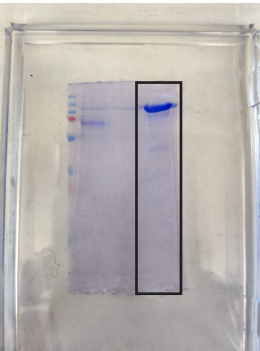

Supplement: SourceData FS1 — is the source file for Fig. S1. [file JCB_202206132_SourceDataFS1.pdf]

# Source Data SF3

$\alpha$ -CGB

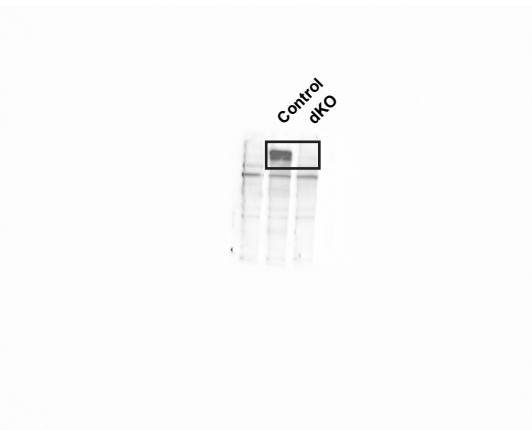

Membrane ( $\alpha$ -CGB)

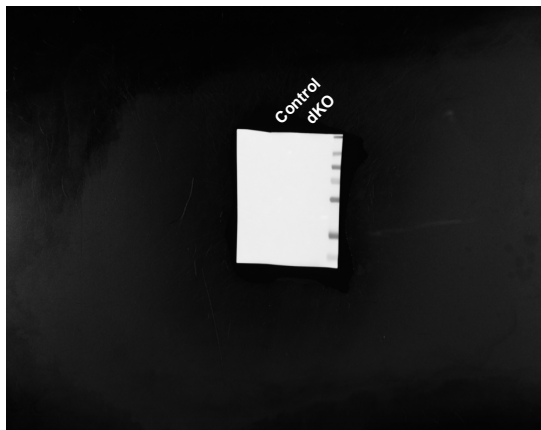

$\alpha$ -Actin

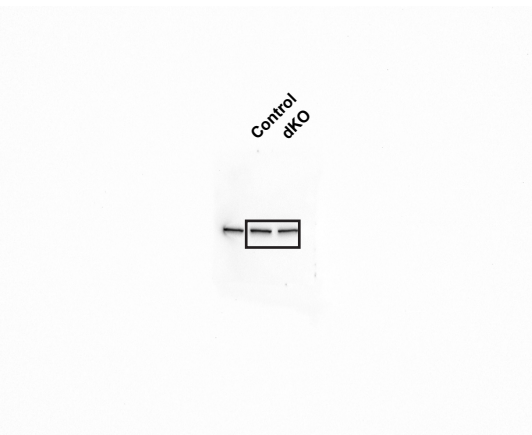

Membrane ( $\alpha$ -Actin)

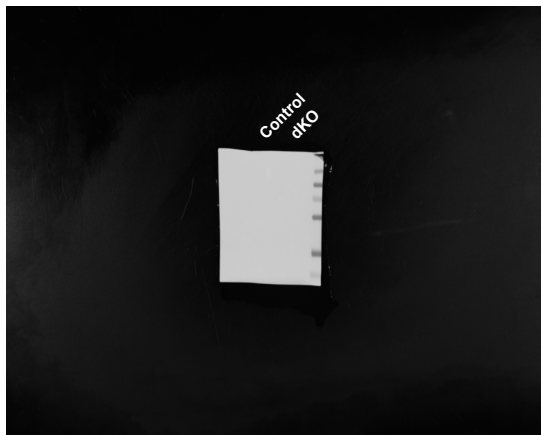

Supplement: SourceData FS3 — is the source file for Fig. S3. [file JCB_202206132_SourceDataFS3.pdf]

# Source Data SF5

$\alpha$ -GFP

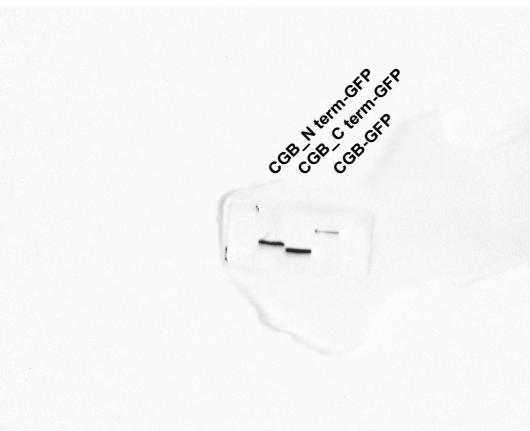

Membrane ( $\alpha$ -GFP)

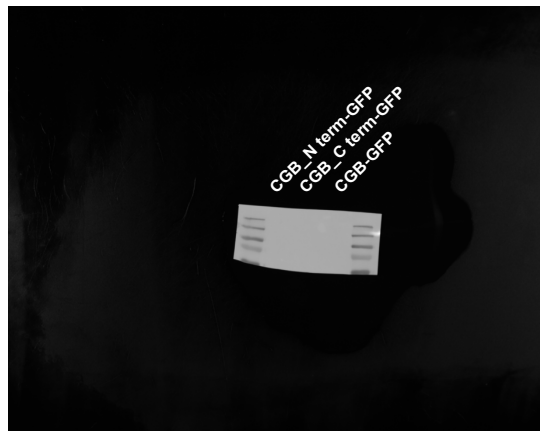

$\alpha$ -Actin

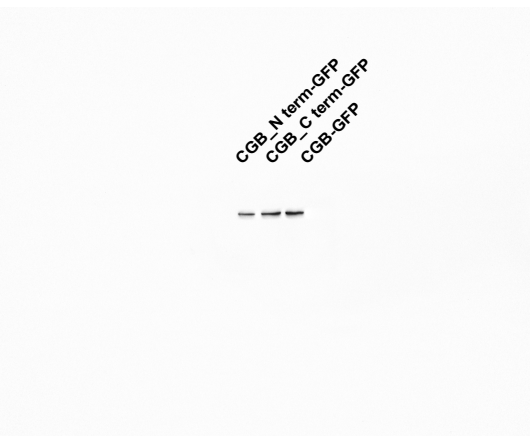

Membrane ( $\alpha$ -Actin)

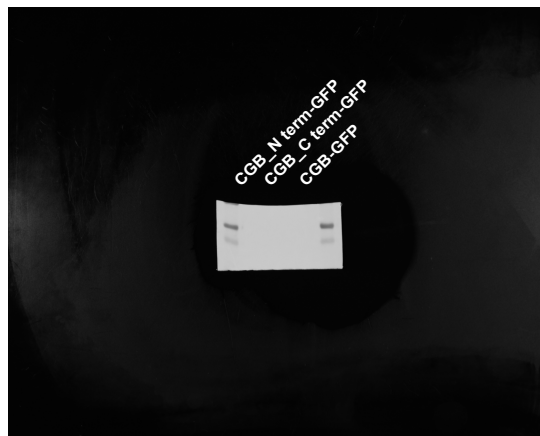

Supplement: SourceData FS5 — is the source file for Fig. S5. [file JCB_202206132_SourceDataFS5.pdf]
