# Supplementary material for: Liquid–liquid phase separation facilitates the biogenesis of secretory storage granules
Source: J Cell Biol. 2022 Sep 29;221(12):e202206132. doi: 10.1083/jcb.202206132 (PMC9526250; doi:10.1083/jcb.202206132)

Source Data F4

α–GFP

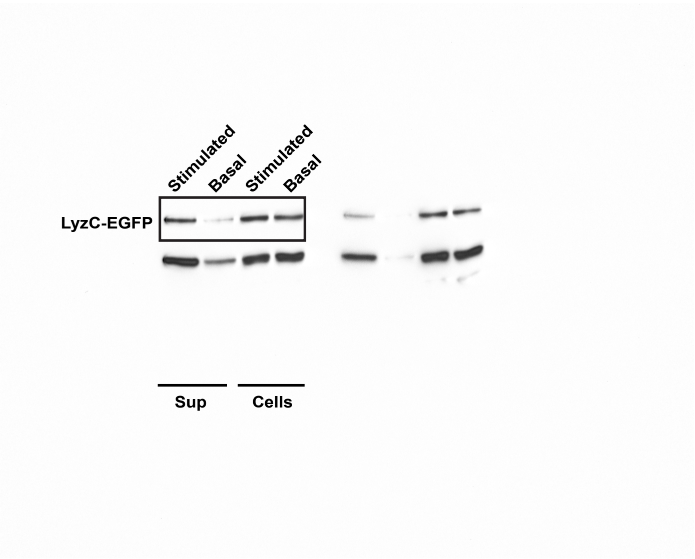

Membrane (α–GFP)

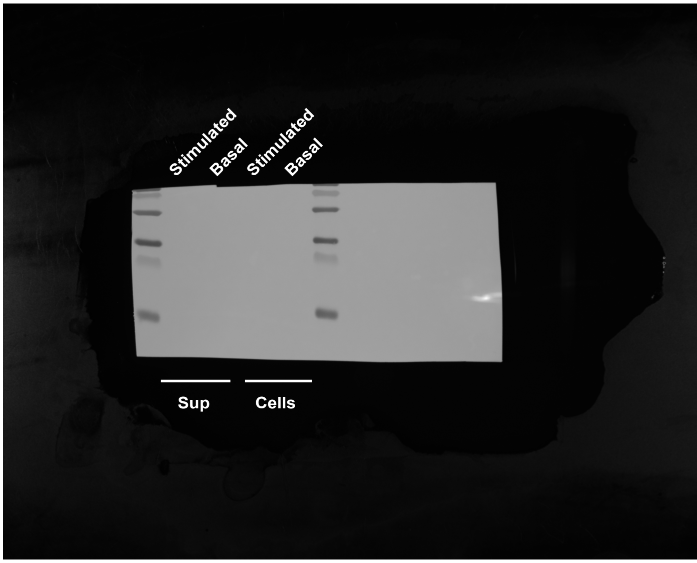

α–SNAP tag

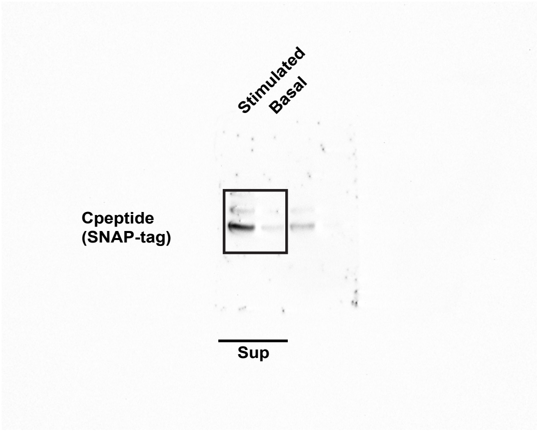

Membrane (α–SNAP tag)

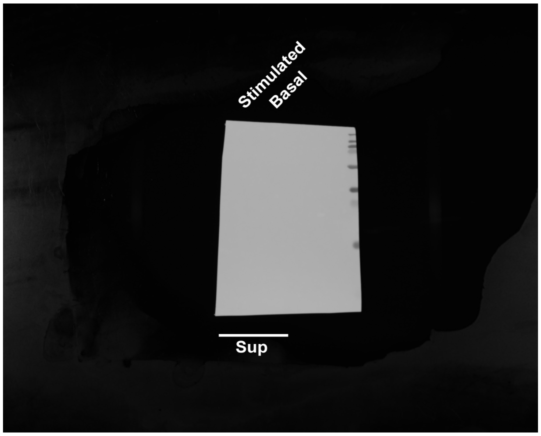

α–Actin

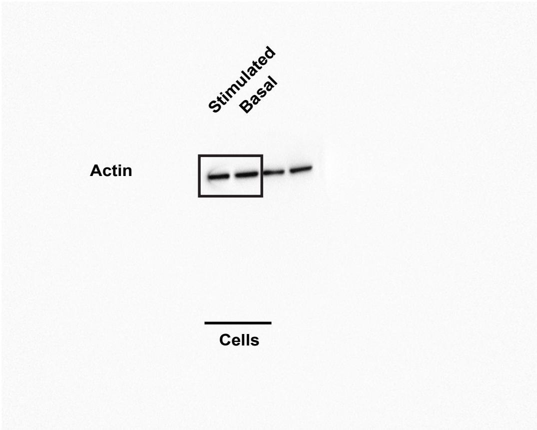

Membrane (α–Actin)

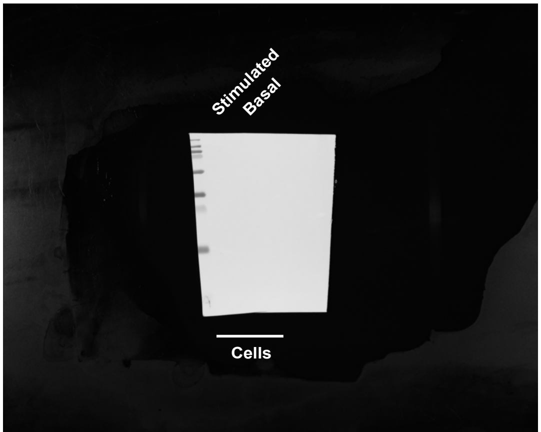

Supplement: SourceData F4 — is the source file for Fig. 4. [file JCB_202206132_SourceDataF4.pdf]
